# Similarities and differences between variants called with human reference genome HG19 or HG38

**DOI:** 10.1186/s12859-019-2620-0

**Published:** 2019-03-14

**Authors:** Bohu Pan, Rebecca Kusko, Wenming Xiao, Yuanting Zheng, Zhichao Liu, Chunlin Xiao, Sugunadevi Sakkiah, Wenjing Guo, Ping Gong, Chaoyang Zhang, Weigong Ge, Leming Shi, Weida Tong, Huixiao Hong

**Affiliations:** 1 Division of Bioinformatics and Biostatistics, National Center for Toxicological Research, U.S. Food and Drug Administration, Jefferson, AR 72079 USA; 2 Immuneering Corporation, Cambridge, MA 02142 USA; 3 Center for Pharmacogenomics, Fudan University, Shanghai, China; 4 National Center for Biotechnological Information, National Institutes of Health, Bethesda, MD 20894 USA; 5 Environmental Laboratory, US Army Engineer Research and Development Center, Vicksburg, MS 39180 USA; 6 School of Computing, The University of Southern Mississippi, Hattiesburg, MS 39406 USA

**Keywords:** Next generation sequencing, Human reference genomes, SNV, Calling pipeline comparison

## Abstract

**Background:**

Reference genome selection is a prerequisite for successful analysis of next generation sequencing (NGS) data. Current practice employs one of the two most recent human reference genome versions: HG19 or HG38. To date, the impact of genome version on SNV identification has not been rigorously assessed.

**Methods:**

We conducted analysis comparing the SNVs identified based on HG19 vs HG38, leveraging whole genome sequencing (WGS) data from the genome-in-a-bottle (GIAB) project. First, SNVs were called using 26 different bioinformatics pipelines with either HG19 or HG38. Next, two tools were used to convert the called SNVs between HG19 and HG38. Lastly we calculated conversion rates, analyzed discordant rates between SNVs called with HG19 or HG38, and characterized the discordant SNVs.

**Results:**

The conversion rates from HG38 to HG19 (average 95%) were lower than the conversion rates from HG19 to HG38 (average 99%). The conversion rates varied slightly among the various calling pipelines. Around 1.5% SNVs were discordantly converted between HG19 or HG38. The conversions from HG38 to HG19 had more SNVs which failed conversion and more discordant SNVs than the opposite conversion (HG19 to HG38). Most of the discordant SNVs had low read depth, were low confidence SNVs as defined by GIAB, and/or were predominated by G/C alleles (52% observed versus 42% expected).

**Conclusion:**

A significant number of SNVs could not be converted between HG19 and HG38. Based on careful review of our comparisons, we recommend HG38 (the newer version) for NGS SNV analysis. To summarize, our findings suggest caution when translating identified SNVs between different versions of the human reference genome.

**Electronic supplementary material:**

The online version of this article (10.1186/s12859-019-2620-0) contains supplementary material, which is available to authorized users.

## Background

Next generation sequencing (NGS), especially human whole genome sequencing (WGS), enables precision medicine and provides a bais for population genetics by directly querying the genetic architecture of individuals with single nucleotide resolution [1]. NGS technology empowers researchers to extract meaningful genetic information from a genome rapidly, which is a driving imperative for the success of clinical applications [2–5]. For example, genetic variants associated with human disease risk could be pinpointed via NGS analysis, accelerating successful diagnosis or precision treatment identification [6–8].

Single nucleotide variant (SNV) detection and genotype determination are paramount to the success of genetic studies [9]. Successful bioinformatic analysis plays a key role in NGS data interpretation [10–12]. Most bioinformatics approaches rely on alignment [13], a step where short sequencing reads from a sequencing platform are mapped to the long string of the reference genome. After the first version of the human genome was published [14, 15], subsequent incremental improvements on the human genome have been released thus and today many versions of the human genome exist. Since different human reference genome versions currently are in use [16], assessing and understanding concordance between genetic variants detected using different reference genomes is important for successful translation of NGS findings into clinically actionable discoveries. Historically, the newest release is recommended by the community for its accuracy [17]. However, to build on previous research results or make a current study comparable to results obtained using previous human reference genome versions [18–20], genetic variants obtained from one reference genome version must sometimes be converted to another older version.

To address this common challenge, several tools have been developed for converting between different human genome versions [16]. However, the concordance between the genetic variants obtained using one version versus those converted from another version has not been assessed to date. The human reference genome hs37d5 (ftp://ftp-trace.ncbi.nlm.nih.gov/1000genomes/ftp/ technical/reference/phase2_reference_assembly_sequence/hs37d5.fa.gz) (termed as HG19 hereafter) and GRCh38 (ftp://ftp.ncbi.nlm.nih.gov/genomes/all/GCA/000/001/405/ GCA_000001405.15_GRCh38/seqs_for_alignment_pipelines.ucsc_ids/GCA_000001405.15_GRCh38_no_alt_plus_hs38d1_analysis_set.fna.gz) (termed as HG38 hereafter) are by far the two most widely used versions of the human reference genome in WGS data analysis in 2018. Therefore, we conducted analysis comparing SNVs identified in GIAB WGS data [21, 22] using HG19 or HG38 to assess the consistency between these two versions of human reference genome. SNVs were called using twenty-six different pipelines with alignments to HG19 or HG38. Two conversion tools (Picard [23] (http://broadinstitute.github.io/picard/) and CrossMap [23]) were utilized to convert between HG19 and HG38. The conversion rate and discordant rate in SNVs generated using HG19 or HG38 were calculated. The characteristics of the discordant SNVs were studied and detailed herein.

## Methods

### Study design

**Fig. 1:**
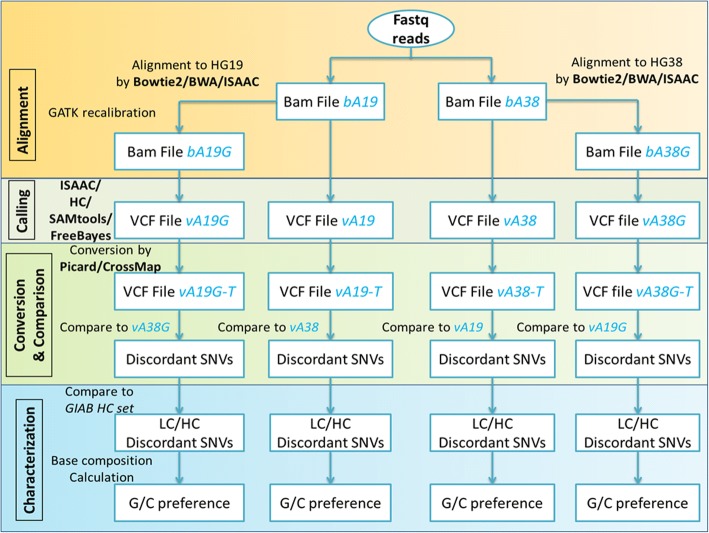
Study design. Whole genome sequencing data from GIAB reference sample NA12878 was downloaded and aligned to human genomes HG19 and HG38 using three aligners followed by SNVs calling using various calling algorithms. The SNVs were then converted between the two reference genomes using Picard and CrossMap. To pinpoint discordant SNVs, converted SNVs were compared against SNVs identified by directly using the target reference genome version. Finally, discordant SNVs were characterized by read depth, low-confidence frequency and prevalence of G/C reference alleles

This study consists of four main phases:

1. Reads were aligned to HG19 or HG38 using three aligners (Bowtie2, BWA, ISAAC).
2. SNVs were called using three algorithms (FreeBayes, GATK HaplotypeCaller (HC), ISAAC, SAMtools).
3. Coordinates of SNVs were converted between HG19 and HG38 using two tools (Picard, CrossMap).
4. Comparative analysis was conducted on the converted SNVs from step 3 (Fig. 1).

First, the raw reads downloaded from GIAB were aligned to human reference genomes HG19 and HG38 separately using three popular aligners (BWA-mem [24], Bowtie2 [25] and ISAAC [26]). For each raw BAM file from each alignment, a GATK recalibration BAM file was generated following the GATK community recommended guidelines [27, 28]. For each of the four BAM files from each aligner, three calling algorithms (ISAAC [26], HC [28], SAMtools [29] and FreeBayes [30]) were used to call SNVs. Next, 26 sets of SNVs (24 generated using above described pipelines and 2 downloaded from GIAB) from HG19 (termed as 38HG19_SNVs hereafter) were converted to SNVs corresponding to HG38 (termed as 19HG38_SNVs hereafter). Similarly, the 26 sets of SNVs from HG38 (termed as 19HG38_SNVs hereafter) were converted to HG19 (termed as 38HG19_SNVs hereafter). The SNV conversions between HG38 and HG19 were performed using LiftoverVcf from the Picard package (http://broadinstitute.github.io/picard/) (Picard is used hereafter) and CrossMap [23]. Then, comparisons between HG19_SNVs and 38HG19_SNVs and between HG38_SNVs and 19HG38_SNVs based on genome position and genotype information were conducted with in-house perl scripts. The discordant SNVs were evaluated for read depth and annotated as being GIAB low and high confidence regions. Lastly, the discordant SNVs were partitioned by the four reference alleles to examine their G/C balance.

### Data downloaded

#### Sequencing data from GIAB

The WGS data from CEPH/HapMap sample NA12878 from the GIAB project [21] was downloaded from ftp://ftp-trace.ncbi.nlm.nih.gov/giab/ftp/data/NA12878/NIST_NA12878_HG001_HiSeq_300x/. The raw reads at ~300X were from the Illumina HiSeq 2500 using paired-end library with 148 base pairs (bp) in read length.

#### SNVs from GIAB

SNVs in GIAB’s Novoalign aligned HG19 and HG38 data were called using HC v3.5 [31] and FreeBayes 0.9.20 [30]. These sets of SNVs were downloaded from GIAB (ftp://ftp-trace.ncbi.nlm.nih.gov/giab/ftp/release/NA12878_HG001/latest).

### Alignment

The raw reads were aligned to HG19 and HG38. Three aligners were used: BWA (v0.7.15) [24], Bowtie2 (v2.2.9) [25] and ISAAC (v01.15.04.01) [26]. For BWA, default parameters were used with a minimum seed length of 19 bp. For Bowtie2, the length of seed substrings was set at 22 bp with 0 bp mismatch in the seed allowed. For ISAAC, the seed length was set at 32 bp. The alignment rate for each aligner was calculated by SAMtools (v2.3.0) [32] based on the aligner produced BAM files. All alignment tasks were run in parallel on the local cluster at National Center for Toxicological Research.

### GATK recalibration

The GATK best practices workflow recommends GATK post-processing recalibration [28], which is believed to improve variant calling accuracy. In addition to the BAM files yielded from alignment, we generated additional BAM files by applying duplicate marking, local realignment around indels and base quality score recalibration for SNV calling. First, duplicates were marked by the MarkDuplicates and AddOrReplaceReadGroups commands from Picard tools (v2.7.1, http://broadinstitute.github.io/picard/). Subsequently, local realignment around known indels was done by the IndelRealigner command in GATK (v3.7) [28] with known indels from the 1000 genome project for both HG19 and HG38. Lastly, the BaseRecalibrator and PrintReads commands in GATK were run to apply base calibration. Both the recalibrated BAM files and the original BAM files were used for SNV calling.

### SNV calling

Three different callers (FreeBayes (v1.1.0–1) [30], HC (v3.7) [28], ISAAC (v1.0.7) [26], SAMtools (v1.3.1) [29]) were run on the BAM files to call SNVs. Option “-X -0 -u -v” was utilized for FreeBayes. For HC, the minimum phred-scaled confidence threshold at which variants should be called was set at 30 and reads with wonky CIGAR strings were removed by “-rf BadCigar”. For ISAAC, SNV calling was run with a minimum MAPQ score equal to 20 and a minimum genotype score less than 30 as filters. SNVs were called by SAMtools using the mpileup and bcftools (v1.3.1) [33] commands. Therefore, we called SNVs using the 20 pipelines listed in Table 1, using either HG19 or HG38.

**Table 1:** The 26 SNV calling pipelines

Number	Aligner	GATK recalibration	Caller
1	Novoalign	N	FreeBayes
2	Novoalign	N	HC
3	ISAAC	N	FreeBayes
4	ISAAC	Y	FreeBayes
5	ISAAC	N	HC
6	ISAAC	Y	HC
7	ISAAC	N	ISAAC
8	ISAAC	Y	ISAAC
9	ISAAC	N	SAMtools
10	ISAAC	Y	SAMtools
11	BWA	N	FreeBayes
12	BWA	Y	FreeBayes
13	BWA	N	HC
14	BWA	Y	HC
15	BWA	N	ISAAC
16	BWA	Y	ISAAC
17	BWA	N	SAMtools
18	BWA	Y	SAMtools
19	Bowtie2	N	FreeBayes
20	Bowtie2	Y	FreeBayes
21	Bowtie2	N	HC
22	Bowtie2	Y	HC
23	Bowtie2	N	ISAAC
24	Bowtie2	Y	ISAAC
25	Bowtie2	N	SAMtools
26	Bowtie2	Y	SAMtools

### VCF file processing

Indels and structural variants such as copy number variations are not as reproducible as SNVs [34, 35], therefore indel and structural variant concordance from different versions of the human reference genome is not assessed here. Only SNVs from each call set were kept for assessment of reference genome version impact. Vcftools (v0.1.15) [36] was used to remove indels from the VCF files prior to the comparative analyses.

### SNV conversion

Coordinate conversion is needed for SNV comparison between two different versions of the reference genome. Two conversion tools were used to convert SNVs between HG19 and HG38. The first tool is Picard (LiftoverVcf command from Picard, v2.7.1, http://broadinstitute.github.io/picard/). The second tool is a standalone program named CrossMap [23]. Both tools are popular and request a fasta file of reference genome and chain file as common inputs. A chain file is a text file defined by UCSC which records chromosomal coordinate relationships between different genomes [16].

### Conversion rate calculation

The conversion efficiency was assessed using conversion rates which were calculated for HG19 to HG38 by Picard and CrossMap using Eqs. (1) and (2), respectively, and for HG38 to HG19 by Picard and CrossMap using Eqs. (3) and (4), respectively, for all 40 sets of SNV calls.

1 $$ {CR}_{Picard}^{HG19}=100\frac{19 HG38\_{SNVs}_{Picard}}{HG19\_ SNVs} $$

2 $$ {CR}_{CrossMap}^{HG19}=100\frac{19 HG38\_{SNVs}_{CrossMap}}{HG19\_ SNVs} $$

3 $$ {CR}_{Picard}^{HG38}=100\frac{38 HG19\_{SNVs}_{Picard}}{HG38\_ SNVs} $$

4 $$ {CR}_{CrossMap}^{HG38}=100\frac{38 HG19\_{SNVs}_{CrossMap}}{HG38\_ SNVs} $$

$19 HG38\_{SNVs}_{Picard}$ and $19 HG38\_{SNVs}_{CrossMap}$ indicate SNVs called with HG19 that were successfully converted to corresponding positions in HG38 using conversion tools Picard and CrossMap, respectively. $38 HG19\_{SNVs}_{Picard}$ and $38 HG19\_{SNVs}_{CrossMap}$ represent the SNVs called with HG38 that were successfully converted to corresponding positions in HG19 using conversion tools Picard and CrossMap, respectively.

### Discordant rate calculation

The converted SNVs which are different from the directly called SNVs were identified based on their positions in the genome only or both their positions and genotypes. They are named position discordant (PD) SNVs and genotype discordant (GD) SNVs by the target reference genome and by the conversion tool, ($PD\_{SNVs}_{tool}^{genome}$) and ($GD\_{SNVs}_{tool}^{genome}$), respectively, for each set of SNVs. Position discordant rate (${PDR}_{tool}^{genome}$) and genotype discordant rate (${GDR}_{tool}^{genome}$) are the percentages of the position discordant SNVs and the genotype discordant SNVs among the converted SNVs and were calculated using Eqs. (5) to (8) and (9) to (12), respectively.

5 $$ { PD R}_{Picard}^{HG19}=100\frac{PD\_{SNVs}_{Picard}^{HG19}}{38 HG19\_{SNVs}_{Picard}} $$

6 $$ { PD R}_{CrossMap}^{HG19}=100\frac{PD\_{SNVs}_{CrossMap}^{HG19}}{38 HG19\_{SNVs}_{CrossMap}} $$

7 $$ { PD R}_{Picard}^{HG38}=100\frac{PD\_{SNVs}_{Picard}^{HG38}}{19 HG38\_{SNVs}_{Picard}} $$

8 $$ { PD R}_{CrossMap}^{HG38}=100\frac{PD\_{SNVs}_{CrossMap}^{HG38}}{19 HG38\_{SNVs}_{CrossMap}} $$

9 $$ { GD R}_{Picard}^{HG19}=100\frac{GD\_{SNVs}_{Picard}^{HG19}}{38 HG19\_{SNVs}_{Picard}} $$

10 $$ { GD R}_{CrossMap}^{HG19}=100\frac{GD\_{SNVs}_{CrossMap}^{HG19}}{38 HG19\_{SNVs}_{CrossMap}} $$

11 $$ { GD R}_{Picard}^{HG38}=100\frac{GD\_{SNVs}_{Picard}^{HG38}}{19 HG38\_{SNVs}_{Picard}} $$

12 $$ { GD R}_{CrossMap}^{HG38}=100\frac{GD\_{SNVs}_{CrossMap}^{HG38}}{19 HG38\_{SNVs}_{CrossMap}} $$

Using the results from BWA alignment and SAMtools calling as an example, two sets of SNVs were obtained, one from the alignment onto HG19 (HG19_SNVs) and the other from the alignment onto HG38 (HG38_SNVs). To convert HG19_SNVs to the positions in HG38, the HG38 equivalent SNVs $19 HG38\_{SNVs}_{Picard}$ and $19 HG38\_{SNVs}_{CrossMap}$ were generated using Picard and CrossMap, respectively. The conversion rates for HG19_SNVs were then calculated using Eqs. (1) and (2). In a similar way the conversion rates for HG38_SNVs were calculated using Eqs. (3) and (4). Thereafter, $19 HG38\_{SNVs}_{Picard}$ and $19 HG38\_{SNVs}_{CrossMap}$ were compared with HG38_SNVs to find the position discordant SNVs ($PD\_{SNVs}_{Picard}^{HG38}$ and $PD\_{SNVs}_{CrossMap}^{HG38}$) and the genotype discordant SNVs ($GD\_{SNVs}_{Picard}^{HG38}$ and $GD\_{SNVs}_{CrossMap}^{HG38}$). Then the position discordant rates and genotype discordant rates were calculated using Eqs. (7–8) and Eqs. (11–12), respectively. In an analogous way, the discordant rates for the converted SNVs from HG38 to HG19 were calculated.

### Discordant SNV characterization

To characterize discordant SNVs, we first divided the position discordant (PD) SNVs and genotype discordant (GD) SNVs by target genome (TG) version (HG19 or HG38) and by conversion tool (Picard or CrossMap): $PD\_{SNVs}_{tool}^{TG}$ and $GD\_{SNVs}_{tool}^{TG}$. They were further divided into high-confidence (HC) SNVs (${}^{HC} PD\_{SNVs}_{tool}^{TG}$ and ${}^{HC} GD\_{SNVs}_{tool}^{TG}$) and low-confidence (LC) SNVs (${}^{LC} PD\_{SNVs}_{tool}^{TG}$ and ${}^{LC} GD\_{SNVs}_{tool}^{TG}$) using the HC/LC SNVs determined by GIAB. In the same way, all SNVs that were converted to a TG using a conversion tool (${SNVs}_{tool}^{TG}$) were divided into ${}^{HC}{SNVs}_{tool}^{TG}$ and ${}^{LC}{SNVs}_{tool}^{TG}$. We then compared the distributions of HC and LC SNVs by the logarithmic values of the ratios calculated using eqs. (13) and (14) for the position discordant SNVs ratio (PR) and genotype discordant SNVs ratio (GR), respectively.

13 $$ PR=\raisebox{1ex}{$\frac{{}^{LC} PD\_{SNVs}_{tool}^{TG}}{{}^{LC}{SNVs}_{tool}^{TG}}$}\!\left/ \!\raisebox{-1ex}{$\frac{{}^{HC} PD\_{SNVs}_{tool}^{TG}}{{}^{HC}{SNVs}_{tool}^{TG}}$}\right. $$

14 $$ GR=\raisebox{1ex}{$\frac{{}^{LC} GD\_{SNVs}_{tool}^{TG}}{{}^{LC}{SNVs}_{tool}^{TG}}$}\!\left/ \!\raisebox{-1ex}{$\frac{{}^{HC} GD\_{SNVs}_{tool}^{TG}}{{}^{HC}{SNVs}_{tool}^{TG}}$}\right. $$

We also compared the percentage of reference allele G and C between the discordant SNVs for $PD\_{SNVs}_{tool}^{TG}$ and $GD\_{SNVs}_{tool}^{TG}$ using an in-house Perl script. All source data and scripts are provided in the Additional file 1.

## Results and discussion

### HG19 and HG38 produce substantially different alignments

The alignment rates of the three aligners run with HG19 and HG38 are listed in Table 2. The reads aligned well to both HG19 and HG38, indicating high quality sequencing data from GIAB and ensuring credibility of the produced BAM files for subsequent SNV calling. BWA-mem had the highest alignment rate, and ISAAC and Bowtie2 were tied at a slightly lower alignment rate. No significant difference in overall alignment rates between HG19 and HG38 was observed across the three aligners.

**Table 2:** Alignment rates between genome versions and aligners

	HG19	HG38
Aligners	Alignment Rate (%)	Alignment Rate (%)
Bowtie2	98.559	98.503
BWA	99.629	99.633
ISAAC	99.146	99.034

However, read coverage across reference genome alignments showed significant differences between HG19 and HG38. Around 6.5% of the bases in HG19 and 4.4% of the bases in HG38 had no reads aligned by any of the three aligners (Additional file 2: Table S1). Not surprisingly, the newer genome version (HG38) had better genome coverage than the older version (HG19), suggesting preference for the newer version when undergoing sequence analysis. The three aligners produced very similar genome coverages. Thus, aligner selection may be a more minor concern in sequencing data analysis compared to reference genome selection. We further calculated coverage for all bases in all alignment results and examined the coverage distribution from 1 read to 600 reads (Additional file 2: Table S1).

**Fig. 2:**
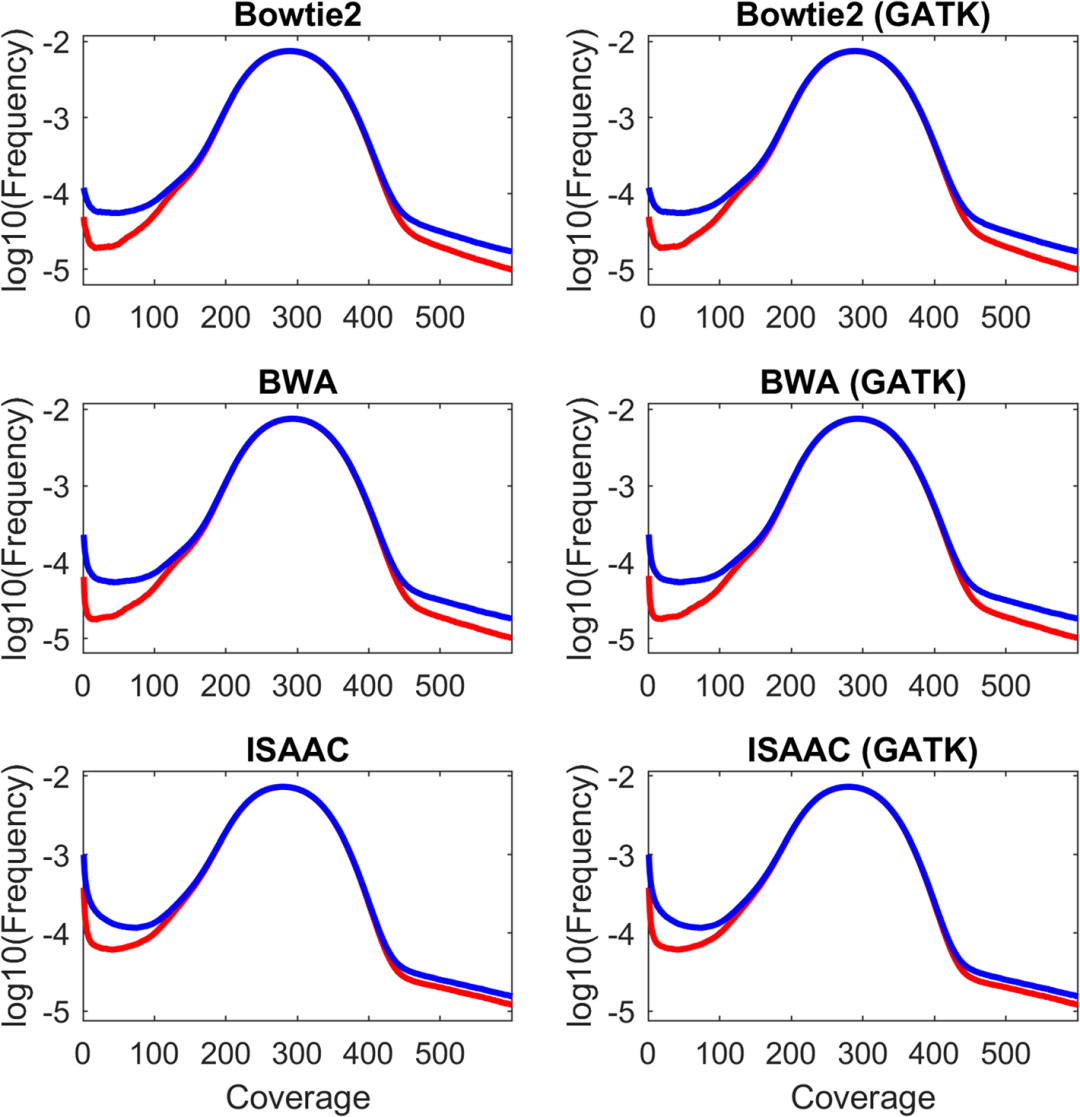
Distribution of genomic coverage. The coverage from each pipeline is plotted against frequency in a log scale with HG19 as red lines and HG38 as blue lines. The two sub-figures in each row are a specific aligner depicted by the titles above the sub-figures. The three sub-figures in the left panel are alignment results without GATK realignment while the right panel contains alignment results with GATK realignment

The coverage from each pipeline is plotted against frequency in a log scale with HG19 as red lines and HG38 as blue lines in Fig. 2. The distribution of coverage for HG19 and HG38 showed significant differences for all alignments, as evidenced by t-test *p*-values less than 0.05 for BWA-mem and Bowtie2 and slightly larger than 0.05 for ISAAC (Additional file 2: Table S2). More interestingly, the differences came from both low and high coverage distributions. For the bases with coverage close to the sequencing depth (300X), HG19 and HG38 performed equally well. In other words, for genomic regions with intermediate amounts of aligned reads, the two versions of human genome were aligned equally. However, for genomic regions with very high or very low amounts of aligned reads, HG19 and HG38 more often produced different alignments. The regions with too few or too many mapped reads are typically difficult regions to be assembled and often assemblies of these areas need improvement. Therefore, the alignment results for the two versions of reference genome might be caused by the improvements of HG38 in these challenging regions. Translating SNVs identified from very low or high coverage regions between genome versions merits caution.

Comparison of coverage distribution between alignments without and with GATK realignment (Additional file 3: Figure S1) revealed that GATK realignment had very little impact. The t-test p-values were close to 1 (Additional file 2: Table S2) and Pearson correlation coefficients were close to 1 (Additional file 2: Table S3). The coverage distribution for different aligners was compared in Additional file 3: Figure S2. BWA-mem and Bowtie2 performed almost identically. In contrast, ISAAC performed differently from the other two aligners in low coverage genomic regions. Even though genomic coverage for different aligners was not significantly different, our comparative analysis of coverage distribution revealed that genetic variants detected in low coverage genomic regions from different aligners should be carefully inspected.

### Calling with HG38 generated more SNVs than calling with HG19

**Fig. 3:**
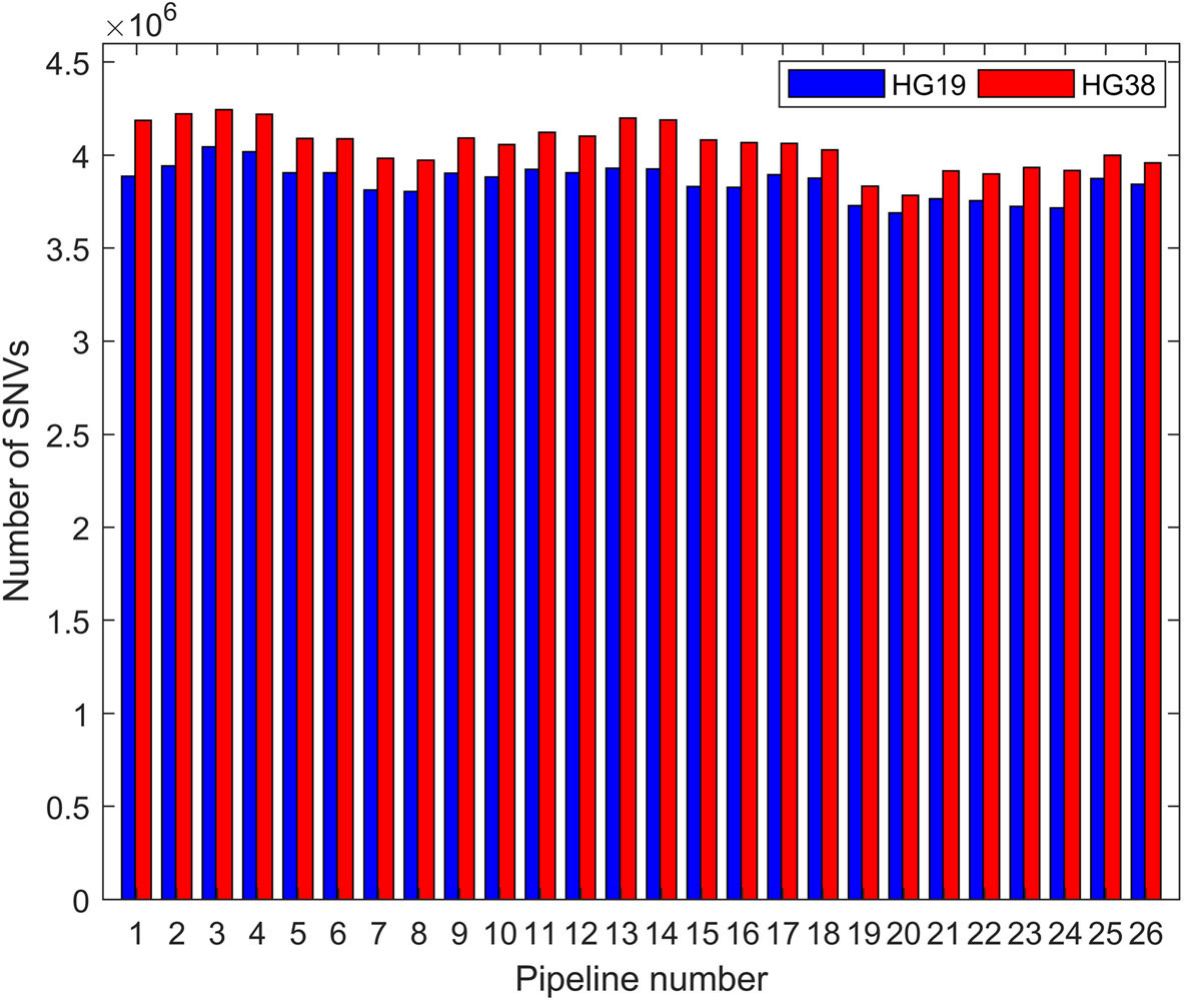
SNVs called from different pipelines. Numbers of SNVs (y-axis) is plotted as bar height. The x-axis contains pipeline numbers found in Table 1. The blue bars represent HG19 alignments and the red bars represent HG38 alignments

The sequencing data was aligned to HG19 and HG38 using three alignment tools followed by GATK realignment. The alignment results, with and without realignment, were used to call SNVs using three algorithms, resulting in 48 sets of SNV calls. We also downloaded four sets of SNVs from GIAB which were obtained from HC and FreeBayes with alignments to HG19 and HG38 using Novoalign. The number of SNVs is plotted in Fig. 3 for all 52 sets of SNVs. No significant variation in the numbers of SNVs was found comparing the three aligners. FreeBayes yielded slightly more SNVs than HC and SAMtools, which identified slightly more SNVs than ISAAC. However, the numbers of SNVs identified from the alignments to the newer version of the human genome (HG38, red bars) are significantly larger (by about 5%) than those detected from the alignments to the older version (HG19, blue bars) for otherwise identical pipelines. On average, 3,859,100 SNVs were called from alignment to HG19 and 4,048,565 SNVs were identified from alignment to HG38, in agreement with what has been previously reported [37]. Here, the improved reference genome (HG38) increased the number of SNVs identified from identical sequencing data, suggesting that genetic variants missed by using HG19 could be identified using HG38. Therefore, we again recommend the newer version (HG38) for sequencing data analysis aimed at variant calling.

### Conversion from HG38 to HG19 was more error prone than HG19 to HG38

**Fig. 4:**
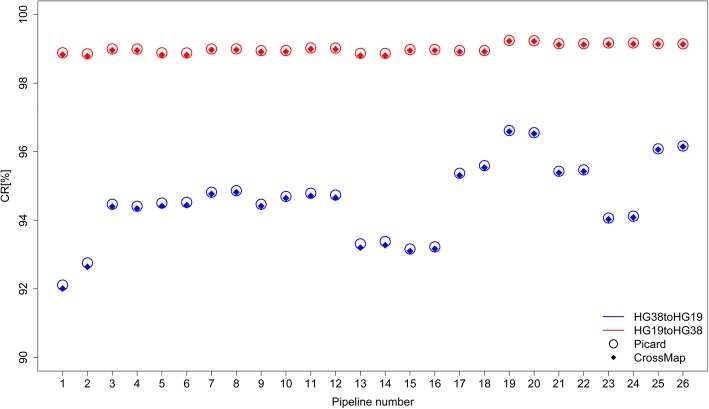
Conversion rates. The conversion rates obtained from Picard are plotted as open circles and the conversion rates yielded from CrossMap are filled diamonds. Results from converting HG38 to HG19 are in blue and results from converting HG19 to HG38 are in red. The x-axis contains pipeline numbers found in Table 1. The y-axis depicts the conversion rates

The conversion rates of all 52 sets of SNVs for the two conversion tools are plotted in Fig. 4. The two conversion tools, Picard (circles) and CrossMap (diamonds), performed nearly identically. Interestingly, the conversion rates from HG19 to HG38 (around 99%) were significantly higher than the corresponding conversion rates from HG38 to HG19 (around 95%). In other words, the coordinates of the older version (HG19) could be readily converted to the coordinate system of the newer version (HG38) while a significant number of SNVs identified from HG38 could not be successfully converted to HG19.

**Fig. 5:**
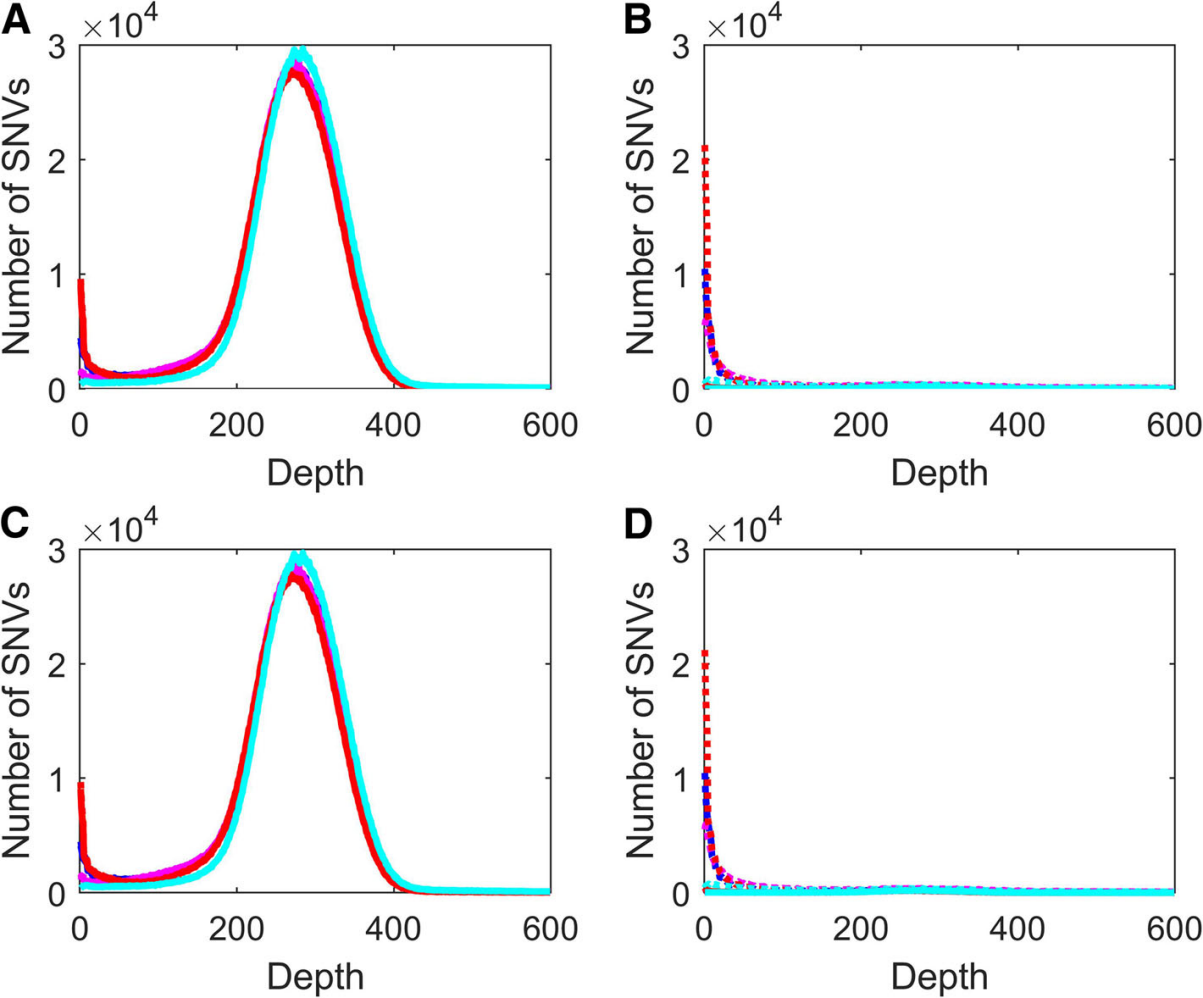
Depth distribution of the converted and not converted SNVs identified from BWA alignment. The number of SNVs (y-axis) is plotted against depth (x-axis) for SNVs called using FreeBayes (blue), HC (magenta), ISAAC (red), and SAMtools (cyan). The solid lines are conversion results from HG19 to HG38. The dotted lines are conversion results from HG38 to HG19. **a** Successfully converted SNVs using CrossMap. **b** SNVs which were not successfully converted using CrossMap. **c** Successfully converted SNVs using Picard. **d** SNVs which were not successfully converted using Picard

We extracted depth information and calculated the frequency of SNVs at each depth (Additional file 2: Table S4). For the SNVs identified from alignments using BWA, no significant variation in SNV depth was found comparing the three calling algorithms or the two conversion tools. Not surprisingly, for the SNVs converted successfully (Fig. 5a and c), the conversions from HG38 to HG19 (dotted lines) were similar with those from HG19 to HG38 (solid lines). However, the depth distributions for the SNVs that were (Fig. 5a and c) or were not (Fig. 5b and d) successfully converted between the two genomes were very different. Strikingly, most of the SNVs that were unable to be converted had very low sequencing depth. Our results demonstrated that some genomic regions, such as repeats, present a challenge for read alignment and are less consistent between different versions of the human genome. Similar observations were obtained for the SNVs identified from alignment results using Bowtie2 and ISAAC (Additional file 2: Table S4). The SNVs with or without GATK realignment showed very similar depth distributions (Additional file 2: Table S4).

### A significant proportion of successfully converted SNVs were discordant

**Fig. 6:**
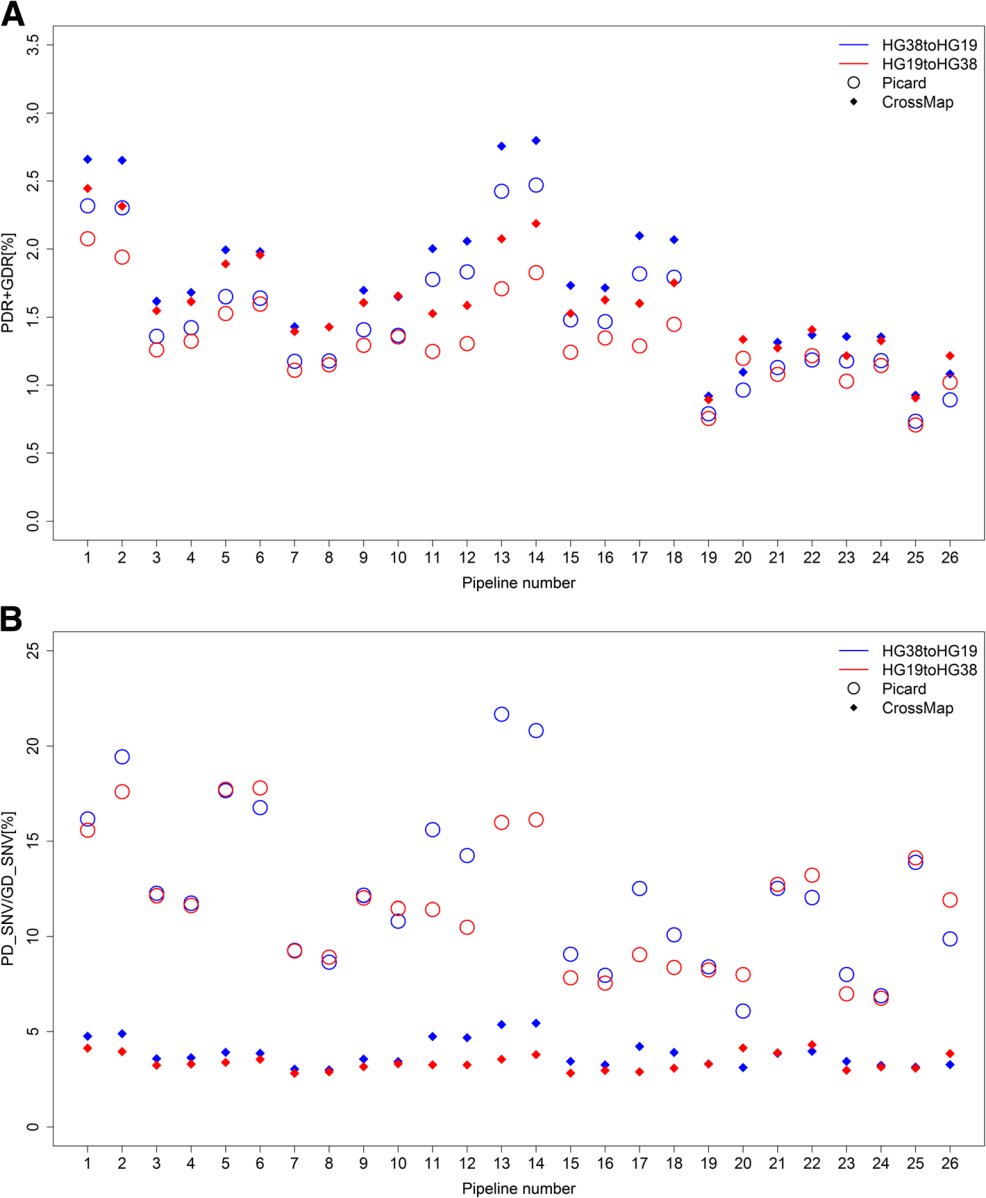
Discordant SNVs. **a** Rates of discordant SNVs in the successfully converted SNVs are portrayed on the y-axis. **b** Ratios of position discordant SNVs to genotype discordant SNVs are depicted on the y-axis. The results from Picard are open circles and the results from CrossMap are filled diamonds. Conversions from HG38 to HG19 are in blue and conversions from HG19 to HG38 are in red. The x-axis contains pipeline numbers from Table 1

The SNVs identified from alignment to a genome version (“source version” hereafter) and converted to another genome version (“target version” hereafter) are expected to also be detected from alignment directly to the target version. However, some of the successfully converted SNVs were not identified from alignment directly to the target version and are termed hereafter as “discordant SNVs”. The rate of discordant SNVs compared to the total of successfully converted SNVs was calculated for all 40 sets of SNVs (Fig. 6a). On average, around 1.5% of successfully converted SNVs were not detected from any alignments directly to the target version. Intriguingly, the discordant rates of the SNVs successfully converted to HG19 (from HG38) were consistently higher than the discordant rates for the SNVs successfully converted to HG38 (from HG19). The SNVs identified from alignment to the newer version (HG38) not only had more SNVs that could not be converted to the older version HG19 (Fig. 4) but also had a higher discordant rate for the converted SNVs compared with the opposite conversion. This result indicates that translation of findings from the newer version (HG38) to the older version (HG19) should be done cautiously. In contrast to the conversion rates, the discordant rates from Picard conversions were consistently lower than the discordant rates from CrossMap conversion across all calling pipelines. The four sets of SNVs downloaded from GIAB yielded discordant rates between 1.94 and 2.66% (pipeline 1 and 2 in Fig. 6a), slightly higher than the discordant rates from the other 36 sets of SNV calls. For the three alignment tools we used, Bowtie2 demonstrated the lowest discordant rates (1.10% ± 0.20%); BWA had the highest discordant rates and highest variation (1.80% ± 0.41%); ISAAC was in the middle (discordant rates 1.51% ± 0.24%).

We next defined properties of the discordant SNVs. The discordant SNVs that were not detected from alignment directly to the target version are hence named “position discordant SNVs”. The discordant SNVs that were identified from alignment to the target version but with different genotypes called are hence named “genotype discordant SNVs”. We first counted numbers of position discordant and genotype discordant SNVs. The ratios of position discordant SNVs to the genotype discordant SNVs were calculated (Fig. 6b). The log2 values of the ratios were much larger than 1, indicating that the majority of the discordant SNVs were position discordant SNVs. Strikingly, CrossMap not only had more discordant SNVs (Fig. 6a) but also yielded more genotype discordant SNVs (lower ratios in Fig. 6b) compared with Picard, which suggests Picard as a superior choice for conversion between different versions of human genome. Unlike the discordant rate, the ratios of position discordant SNVs to genotype discordant SNVs did not show a significant or consistent difference between the two genome conversions considered here. No significant differences were observed between the aligners or the calling algorithms in this regard.

### Discordant SNVs tend to be low confidence calls

**Fig. 7:**
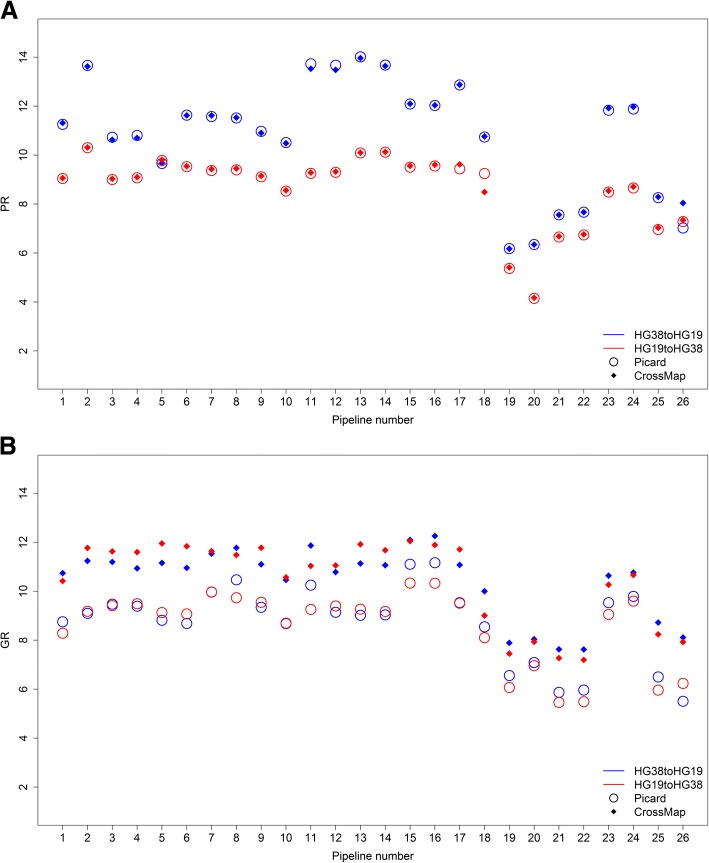
Ratios of LC to HC discordant SNVs. **a** Log2 values of the ratios of position discordant SNVs are on the y-axis. **b** Log2 values of the ratios of genotype discordant SNVs on the y-axis. The results from Picard are open circles and the results from CrossMap are filled diamonds. Conversions from HG38 to HG19 are in blue and conversions from HG19 to HG38 are in red. Numbers along the x-axis come from the pipelines in Table 1

To characterize discordant SNVs, we first compared both the successfully converted SNVs and the discordant SNVs against the GIAB gold standard set of HC and LC SNVs. The ratio of position discordant SNVs and genotype discordant SNV to the converted SNVs were calculated using eqs. (13) and (14) as described in methods. The log2 values of the PR (position discordant ratio) and GR (genotype discordant ratio) for all 40 sets of SNVs are plotted in Fig. 7. Position discordant SNVs (Fig. 7a) and genotype discordant SNVs (Fig. 7b) were more often LC as compared to successfully converted SNVs. Converting from HG38 to HG19 generated significant higher log2 PR values (average of 10.85) than the opposite conversion (average of 8.62) for all 52 sets of SNVs converted using either conversion tool (Fig. 7a). Thus, LC SNVs are a major source of position discordant SNVs and present a HG38 to HG19 conversion challenge. In contrast, no significant differences were found for the GR values when converting either way between HG38 and HG19 (Fig. 7b). Taken together, these results demonstrate that HC and LC SNVs equally contributed to the genotype discordant SNVs relative to the successfully converted SNVs for both conversions. Interestingly, the two conversion tools did not show a significant difference in PR values (Fig. 7a), although CrossMap had consistently higher GR values than Picard (Fig. 7b). The PR and GR values varied substantially between aligners and calling algorithms; but no consistent trend was observed.

### Discordant SNVs are G/C rich

G/C content is well-known to impact SNV calling [38, 39]. To investigate the influence of reference alleles G and C on conversion discordance between HG19 and HG38, we summarized reference allele base composition for discordant SNVs (Additional file 3: Figures S3-S10). The average base composition and standard deviation for the discordant SNVs obtained from conversions of all 52 sets of SNVs was calculated. The discordant SNVs were characterized by each of the four individual bases as well as for the total of G and C together. The nucleotide balance results are listed in Table 3. Importantly, the discordant SNVs had higher GC content (52.24 to 53.86%) compared to the human reference genome GC content rate (42%). This difference between what is expected based on background frequency and what is observed indicates that SNVs with a G/C reference present a more substantial conversion challenge. Our results are consistent with previous literature findings that NGS technology has lower SNV-calling performance on CpG islands [40].

**Table 3:** Base composition of discordant SNVs in percentages (mean ± standard deviation)

Type	Base	Picard		CrossMap	
		HG38➔HG19	HG19➔HG38	HG38➔HG19	HG19➔HG38
Position discordant SNVs	A	23.43 ± 0.78	23.74 ± 0.81	23.32 ± 0.81	23.66 ± 0.80
	T	23.86 ± 0.67	24.02 ± 0.86	23.64 ± 0.70	23.89 ± 0.85
	G	26.21 ± 0.71	25.92 ± 0.8	26.37 ± 0.75	26.06 ± 0.78
	C	26.5 ± 0.81	26.32 ± 0.87	26.62 ± 0.81	26.35 ± 0.89
	G + C	52.71 ± 1.44	52.24 ± 1.64	52.99 ± 1.49	52.41 ± 1.64
Genotype discordant SNVs	A	23.06 ± 1.61	23.07 ± 1.66	23.49 ± 0.88	23.57 ± 0.91
	T	23.11 ± 1.66	23.07 ± 1.75	23.59 ± 0.81	23.67 ± 0.93
	G	26.74 ± 1.65	26.84 ± 1.92	26.79 ± 0.81	26.19 ± 1.16
	C	27.08 ± 1.47	27.02 ± 1.45	26.13 ± 0.92	26.57 ± 0.67
	G + C	53.82 ± 2.93	53.86 ± 3.13	52.92 ± 1.62	52.77 ± 1.73

## Conclusions

We compared SNVs identified using the two most recent versions of the human genome: HG19 and HG38. Alarmingly, a significant proportion of SNVs were not successfully converted (around 5% for SNVs identified using HG38 and 1% for HG19), suggesting that HG38 (the newer version) has some genomic resolution lacking in the older version. Among the successfully converted SNVs, about 1.5% could not be found by alignment directly to the target genome version. Discordant SNVs had lower read depth, were more frequently defined as low confidence by GIAB, and had higher prevalence of reference alleles G and C than concordant SNVs. By these various characteristics, discordant SNVs are deemed to be lower quality. Furthermore, converting SNVs obtained using the newer version (HG38) to the older version (HG19) is more challenging than opposite conversion. Discordant SNVs could be driven by coordinate differences between reference genomes and/or by the conversion tools. Our findings suggest caution when translating genetic findings between different versions of the human reference genome. After carefully reviewing the results of our in depth comparison, we recommend that newer version (HG38) should be used going forward in SNV analysis.

## Additional files

Additional file 1: Perl and shell scripts. This file contains all the scripts for coordinate conversion and SNVs comparison. (PDF 164 kb)

Additional file 2: Supplemental tables. (ZIP 633 kb)

Additional file 3: Supplemental figures. (PDF 900 kb)

## Abbreviations

bp: Base pairs
GD: Genotype discordant
GIAB: Genome-in-a-bottle
GR: Genotype discordant SNVs ratio
HC: HaplotypeCaller
HG19: The human reference genome hs37d5
HG38: The human reference genome GRCh38
LC: Low-confidence
NGS: Next generation sequencing
PD: Position discordant
PR: Position discordant SNVs ratio
SNV: Single nucleotide variant
TG: Target genome
WGS: Whole genome sequencing
