# Correction to: Similarities and differences between variants called with human reference genome HG19 or HG38

**DOI:** 10.1186/s12859-019-2776-7

**Published:** 2019-05-15

**Authors:** Bohu Pan, Rebecca Kusko, Wenming Xiao, Yuanting Zheng, Zhichao Liu, Chunlin Xiao, Sugunadevi Sakkiah, Wenjing Guo, Ping Gong, Chaoyang Zhang, Weigong Ge, Leming Shi, Weida Tong, Huixiao Hong

**Affiliations:** 1 Division of Bioinformatics and Biostatistics, National Center for Toxicological Research, U.S. Food and Drug Administration, Jefferson, AR 72079 USA; 2 Immuneering Corporation, Cambridge, MA 02142 USA; 3 Center for Pharmacogenomics, Fudan University, Shanghai, China; 4 National Center for Biotechnological Information, National Institutes of Health, Bethesda, MD 20894 USA; 5 Environmental Laboratory, US Army Engineer Research and Development Center, Vicksburg, MS 39180 USA; 6 School of Computing, The University of Southern Mississippi, Hattiesburg, MS 39406 USA

**Correction to: BMC Bioinformatics (2019) 20 (Suppl 2): 101**

**https://doi.org/10.1186/s12859-019-2620-0**

After publication of this supplement article [1], it has been brought to our attention that there is information concerning the author Chunlin Xiao missing from the author affiliations and the ‘Acknowledgements’ of the article.

This information is required to comply with NLM/NIH (National Library of Medicine, National Institutes of Health) policies regarding manuscript publication.

As such, with regard to the author affiliations and the ‘Acknowledgements’ respectively, please be advised that:

1. Dr. Chunlin Xiao’s affiliation should include “National Library of Medicine”, making the full affiliation “National Center for Biotechnology Information, National Library of Medicine, National Institutes of Health, Bethesda, Maryland 20894, USA.”

2. The ‘Acknowledgements’ section should include “Dr. Chunlin Xiao was supported by the Intramural Research Program of the National Library of Medicine, National Institutes of Health.”

## References

[1] Pan et al. 2019. Similarities and differences between variants called with human reference genome HG19 or HG38. BMC Bioinformatics*.* 2019, 20 (Suppl 2): 101 DOI: 10.1186/s12859-019-2620-0. 10.1186/s12859-019-2620-0 PMC6419332 30871461

