# Supplementary material for: Similarities and differences between variants called with human reference genome HG19 or HG38
Source: BMC Bioinformatics. 2019 Mar 14;20(Suppl 2):101. doi: 10.1186/s12859-019-2620-0 (PMC6419332; doi:10.1186/s12859-019-2620-0)
Supplement: Supplementary file 1 — Perl and shell scripts. This file contains all the scripts for coordinate conversion and SNVs comparison. (PDF 164 kb) [file 12859_2019_2620_MOESM1_ESM.pdf]

Scripts included in this .pdf file:

(1) Picard conversion and comparison pipeline;
(2) CrossMap conversion and comparison pipeline;
(3) SNV comparison and statistic script referred in pipeline (1) and (2);

```
######## (1) Picard conversion and comparison pipeline #####################

#!/bin/sh

#

#$ -N Picard_TF

#$ -S /bin/bash

#$ -cwd

#$ -j y

#$ -pe multicore 1

#$ -R y

#$ -o /dev/ngs005/scratch/hhong/GIAB/4_1_Picard/log_Picard_TF.chr.log


# datasets

## set path

export JAVA_HOME=/storage2/bgong/tools/jdk1.8.0_91 # JAVA version (build 1.8.0_91-b14)

#export JAVA_HOME=/dev/ngs001/bpan/bin/jdk1.8.0_151/

export ztools=/storage2/zliu/bin



# export tools

export PICARD=$ztools/picard-2.7.1/picard.jar

export RTG=/dev/ngs001/bpan/bin/GIAB_bin/RTG.jar

#export vcfsort=/usr/local/toolbox/libs/vcflib/bin/vcfsort

export vcfsort=/storage2/lwu/software/vcftools_0.1.13/perl/vcf-sort

export bgzip=/dev/ngs001/bpan/bin/GIAB_bin/bgzip

export tabix=/dev/ngs001/bpan/bin/GIAB_bin/tabix
```

GATK=$ztools/GATK3.7/GenomeAnalysisTK.jar

bcftools=$ztools/bcftools-1.3.1

export mybin=$genomewarp:$JAVA_HOME/bin:$ztools/bowtie2-2.2.9:$ztools/GATK3.7:$ztools/bcftools-1.3.1:$ztools/samtools-1.3.1:$ztools/SNVer-0.5.3:$ztools/isaac_variant_caller-1.0.7/etc:$ztools/isaac_variant_caller-1.0.7/bin:$ztools/gvcftools-0.14/bin

export PATH=$mybin:/usr/local/bin:/usr/bin:/bin:/usr/local/sbin:/usr/sbin:/sbin:$PATH

####1.reference transfer###################

#reference fasta file

ref_hg38=/dev/ngs003/bpan/reference_comparison/1_orignal_data/hg38/fasta/GCA_000001405.15_GRCh38_no_alt_plus_hs38d1_analysis_set.fna

ref_hg37=/dev/ngs003/bpan/reference_comparison/1_orignal_data/hg37/fasta/hs37d5.chrAdd.fa

chain file

chainFiledir=/dev/ngs003/bpan/reference_comparison/1_orignal_data/chainFile

chain19to38=$chainFiledir/hg19ToHg38.over.filt.chain

chain38to19=$chainFiledir/hg38ToHg19.over.filt.chain

#query vcf file

hg37_vcf=/dev/ngs005/scratch/hhong/GIAB/3_callingResult/2_SNV_sep/clmrgOut_"$1"_hg37."$2".GATK.snv.recode.cln.chrAdd.vcf

hg38_vcf=/dev/ngs005/scratch/hhong/GIAB/3_callingResult/2_SNV_sep/clmrgOut_"$1"_hg38."$2".GATK.snv.recode.cln.vcf

#workdir

workdir=/dev/ngs005/scratch/hhong/GIAB/4_1_Picard

create tempdir for middle results

mkdir /dev/ngs005/scratch/hhong/GIAB/4_1_Picard/"$1"_"$2"_GATK

outdir=/dev/ngs005/scratch/hhong/GIAB/4_1_Picard/"$1"_"$2"_GATK

mkdir /dev/ngs005/scratch/hhong/GIAB/4_1_Picard/"$1"_"$2"_GATK/temp

tempdir=/dev/ngs005/scratch/hhong/GIAB/4_1_Picard/"$1"_"$2"_GATK/temp

```
#echo $queryvcf

java -Djava.io.tmpdir=$tempdir -Xmx20g -jar $PICARD LiftoverVcf \

I=${hg37_vcf} \

O=$outdir/"$1"_"$2"_GATK_Picard_hg38fr37.chr.vcf \

REJECT=$outdir/rejected_"$1"_"$2"_GATK_hg38fr37_variants.chr.vcf \

CHAIN=${chain19to38} \

R=${ref_hg38}


java -Djava.io.tmpdir=$tempdir -Xmx20g -jar $PICARD LiftoverVcf \

I=${hg38_vcf} \

O=$outdir/"$1"_"$2"_GATK_Picard_hg37fr38.chr.vcf \

REJECT=$outdir/rejected_"$1"_"$2"_GATK_hg37fr38_variants.chr.vcf \

CHAIN=${chain38to19} \

R=${ref_hg37}


####2.HCCcomparison########################

export LD_LIBRARY_PATH=/dev/ngs001/bpan/lib/glibc-2.15/build/:$LD_LIBRARY_PATH

$vcfsort $outdir/"$1"_"$2"_GATK_Picard_hg38fr37.chr.vcf
>$outdir/"$1"_"$2"_GATK_Picard_hg38fr37.chr.sort.vcf

$vcfsort $outdir/"$1"_"$2"_GATK_Picard_hg37fr38.chr.vcf
>$outdir/"$1"_"$2"_GATK_Picard_hg37fr38.chr.sort.vcf

$bgzip -c $outdir/"$1"_"$2"_GATK_Picard_hg38fr37.chr.sort.vcf
>$outdir/"$1"_"$2"_GATK_Picard_hg38fr37.chr.sort.vcf.gz

$bgzip -c $outdir/"$1"_"$2"_GATK_Picard_hg37fr38.chr.sort.vcf
>$outdir/"$1"_"$2"_GATK_Picard_hg37fr38.chr.sort.vcf.gz

$tabix -p vcf $outdir/"$1"_"$2"_GATK_Picard_hg38fr37.chr.sort.vcf.gz

$tabix -p vcf $outdir/"$1"_"$2"_GATK_Picard_hg37fr38.chr.sort.vcf.gz
```

hg38_GIABsnvVCF=$outdir/"$1"_"$2"_GATK_Picard_hg38fr37.chr.sort.vcf.gz

hg37_GIABsnvVCF=$outdir/"$1"_"$2"_GATK_Picard_hg37fr38.chr.sort.vcf.gz

unset LD_LIBRARY_PATH

HCC_hg38_snvVCF=/dev/ngs003/bpan/reference_comparison/1_orignal_data/hg38/HG001_GRCh38_GIAB_highconf_CG-IllFB-IllGATKHC-Ion-10X-SOLID_CHROM1-X_v.3.3.2_highconf_PGandRTGphasetransfer.snv.recode.vcf.gz

HCC_hg37_snvVCF=/dev/ngs003/bpan/reference_comparison/1_orignal_data/hg37/HG001_GRCh37_GIAB_highconf_CG-IllFB-IllGATKHC-Ion-10X-SOLID_CHROM1-X_v.3.3.2_highconf_PGandRTGphasetransfer.snv.recode.chrAdd.vcf.gz

sdf_hg38=/dev/ngs003/bpan/reference_comparison/1_orignal_data/hg38/fasta/hg38sdf

sdf_hg37=/dev/ngs003/bpan/reference_comparison/1_orignal_data/hg37/fasta/hg37sdf_chrAdd

HCCbed_hg38=/dev/ngs003/bpan/reference_comparison/1_orignal_data/hg38/HG001_GRCh38_GIAB_highconf_CG-IllFB-IllGATKHC-Ion-10X-SOLID_CHROM1-X_v.3.3.2_highconf_nosomaticdel_noCENorHET7.bed

HCCbed_hg37=/dev/ngs003/bpan/reference_comparison/1_orignal_data/hg37/HG001_GRCh37_GIAB_highconf_CG-IllFB-IllGATKHC-Ion-10X-SOLID_CHROM1-X_v.3.3.2_highconf_nosomaticdel.chrAdd.bed

outDirCmp_GATKHC_hg38=/dev/ngs005/scratch/hhong/GIAB/4_1_Picard/comp_liftover_hg38_"$1"_"$2"_GATK

outDirCmp_GATKHC_hg37=/dev/ngs005/scratch/hhong/GIAB/4_1_Picard/comp_liftover_hg37_"$1"_"$2"_GATK

```
java -Djava.io.tmpdir=$tempdir -Xmx20g -jar $RTG vcfeval \
    -b "${HCC_hg38_snvVCF}" \
    -c "${hg38_GIABsnvVCF}" \
    -o "${outDirCmp_GATKHC_hg38}" \
    -t "${sdf_hg38}"
```

```
java -Djava.io.tmpdir=$tempdir -Xmx20g -jar $RTG vcffilter \
    -i "${outDirCmp_GATKHC_hg38}"/tp.vcf.gz \
    --include-bed "${HCCbed_hg38}" \
    -o "${outDirCmp_GATKHC_hg38}"/tp_inhighconfbed.vcf.gz

java -Djava.io.tmpdir=$tempdir -Xmx20g -jar $RTG vcfeval \
    -b "${HCC_hg37_snvVCF}" \
    -c "${hg37_GIABsnvVCF}" \
    -o "${outDirCmp_GATKHC_hg37}" \
    -t "${sdf_hg37}"

java -Djava.io.tmpdir=$tempdir -Xmx20g -jar $RTG vcffilter \
    -i "${outDirCmp_GATKHC_hg37}"/tp.vcf.gz \
    --include-bed "${HCCbed_hg37}" \
    -o "${outDirCmp_GATKHC_hg37}"/tp_inhighconfbed.vcf.gz

gzip -d -c "${outDirCmp_GATKHC_hg38}"/tp_inhighconfbed.vcf.gz
>"${outDirCmp_GATKHC_hg38}"/tp_inhighconfbed.vcf

gzip -d -c "${outDirCmp_GATKHC_hg37}"/tp_inhighconfbed.vcf.gz
>"${outDirCmp_GATKHC_hg37}"/tp_inhighconfbed.vcf

cs_cal=/dev/ngs003/bpan/reference_comparison/scripts/cor_VCFvsVCF_v6_HCC_ATGCcnt.pl

perl $cs_cal "${outDirCmp_GATKHC_hg38}"/tp_inhighconfbed.vcf
$outdir/"$1"_"$2"_GATK_Picard_hg38fr37.chr.sort.vcf ${hg38_vcf}
$outdir/CS_Picard_"$1"_"$2"_GATK_hg38TFvshg38.stat.txt
>$outdir/CS_Picard_"$1"_"$2"_GATK_hg38TFvshg38.NCSnotCSpos.txt

perl $cs_cal "${outDirCmp_GATKHC_hg37}"/tp_inhighconfbed.vcf
$outdir/"$1"_"$2"_GATK_Picard_hg37fr38.chr.sort.vcf ${hg37_vcf}
```

```
$outdir/CS_Picard_"$1"_"$2"_GATK_hg37TFvshg37.stat.txt
>$outdir/CS_Picard_"$1"_"$2"_GATK_hg37TFvshg37.NCSnotCSpos.txt
```

######## (2) CrossMap conversion and comparison pipeline #####################

```
#!/bin/sh

#

#$ -N crossmap_TF_GATK

#$ -S /bin/bash

#$ -cwd

#$ -j y

#$ -pe multicore 1

#$ -R y

#$ -o /dev/ngs005/scratch/hhong/GIAB/4_3_crossMap/log_crossmap_TF.GATK.chr.log


# datasets

## set path

export JAVA_HOME=/storage2/bgong/tools/jdk1.8.0_91 # JAVA version (build 1.8.0_91-b14)

#export JAVA_HOME=/dev/ngs001/bpan/bin/jdk1.8.0_151/

export ztools=/storage2/zliu/bin

#export tools

export PICARD=$ztools/picard-2.7.1/picard.jar


export RTG=/dev/ngs001/bpan/bin/GIAB_bin/RTG.jar

#export vcfsort=/usr/local/toolbox/libs/vcflib/bin/vcfsort

export vcfsort=/storage2/lwu/software/vcftools_0.1.13/perl/vcf-sort

export bgzip=/dev/ngs001/bpan/bin/GIAB_bin/bgzip

export tabix=/dev/ngs001/bpan/bin/GIAB_bin/tabix

echo $vcfsort
```

GATK=$ztools/GATK3.7/GenomeAnalysisTK.jar

bcftools=$ztools/bcftools-1.3.1

samtools=$ztools/samtools-1.3.1

CrossMap=/dev/ngs001/bpan/bin/CrossMap-0.2.6/bin/CrossMap.py

export mybin=$JAVA_HOME/bin:$ztools/bowtie2-2.2.9:$ztools/GATK3.7:$ztools/bcftools-1.3.1:$ztools/samtools-1.3.1:$ztools/SNVer-0.5.3:$ztools/isaac_variant_caller-1.0.7/etc:$ztools/isaac_variant_caller-1.0.7/bin:$ztools/gvcftools-0.14/bin

export PATH=$mybin:/usr/local/bin:/usr/bin:/bin:/usr/local/sbin:/usr/sbin:/sbin:$PATH

####1.reference transfer####################

#reference fasta file

ref_hg38=/dev/ngs003/bpan/reference_comparison/1_orignal_data/hg38/fasta/GCA_000001405.15_GRCh38_no_alt_plus_hs38d1_analysis_set.fna

ref_hg37=/dev/ngs003/bpan/reference_comparison/1_orignal_data/hg37/fasta/hs37d5.chrAdd.fa

chain file

chainFiledir=/dev/ngs003/bpan/reference_comparison/1_orignal_data/chainFile

chain19to38=$chainFiledir/hg19ToHg38.over.filt.chain

chain38to19=$chainFiledir/hg38ToHg19.over.filt.chain

#query vcf file

hg37_vcf=/dev/ngs005/scratch/hhong/GIAB/3_callingResult/2_SNV_sep/clmrgOut_"$1"_hg37."$2".GATK.snv.recode.cln.chrAdd.vcf

hg38_vcf=/dev/ngs005/scratch/hhong/GIAB/3_callingResult/2_SNV_sep/clmrgOut_"$1"_hg38."$2".GATK.snv.recode.cln.vcf

#query bed file

hg37_bed=/dev/ngs003/bpan/reference_comparison/1_orignal_data/hg37/supplementaryFiles/inputvcfsandbeds/HG001_GRCh37_CHROM1-X_novoalign_Ilmn150bp300X_GATKHC_gvcf_callable.chrAdd.bed

```
hg38_bed=/dev/ngs003/bpan/reference_comparison/1_orignal_data/hg38/supplementaryFiles/inputvc
fsandbeds/HG001_GRCh38_CHROM1-X_novoalign_150bp300x_GATKHC_gvcf_callable.bed

#outfile

mkdir /dev/ngs005/scratch/hhong/GIAB/4_3_crossMap/"$1"_"$2"_GATK

workdir=/dev/ngs005/scratch/hhong/GIAB/4_3_crossMap/"$1"_"$2"_GATK

outdir=/dev/ngs005/scratch/hhong/GIAB/4_3_crossMap/"$1"_"$2"_GATK

hg38fr37vcf=$workdir/"$1"_"$2"_GATK_crossmap_hg38fr37.chr.vcf

hg38fr37bed=$workdir/"$1"_"$2"_GATK_crossmap_hg38fr37.chr.bed

hg37fr38vcf=$workdir/"$1"_"$2"_GATK_crossmap_hg37fr38.chr.vcf

hg37fr38bed=$workdir/"$1"_"$2"_GATK_crossmap_hg37fr38.chr.bed



# create tempdir for middle results

mkdir /dev/ngs005/scratch/hhong/GIAB/4_3_crossMap/temp

tempdir=/dev/ngs005/scratch/hhong/GIAB/4_3_crossMap/temp


/dev/ngs001/bpan/bin/build_Python-2.7.12/python $CrossMap vcf $chain19to38 $hg37_vcf $ref_hg38
$outdir/"$1"_"$2"_GATK_crossmap_hg38fr37.chr.vcf

/dev/ngs001/bpan/bin/build_Python-2.7.12/python $CrossMap vcf $chain38to19 $hg38_vcf $ref_hg37
$outdir/"$1"_"$2"_GATK_crossmap_hg37fr38.chr.vcf


####2.HCCcomparison#######################

export LD_LIBRARY_PATH=/dev/ngs001/bpan/lib/glibc-2.15/build/:$LD_LIBRARY_PATH

$vcfsort $outdir/"$1"_"$2"_GATK_crossmap_hg38fr37.chr.vcf
>$outdir/"$1"_"$2"_GATK_crossmap_hg38fr37.chr.sort.vcf

$vcfsort $outdir/"$1"_"$2"_GATK_crossmap_hg37fr38.chr.vcf
>$outdir/"$1"_"$2"_GATK_crossmap_hg37fr38.chr.sort.vcf
```

```
$bgzip -c $outdir/"$1"_"$2"_GATK_crossmap_hg38fr37.chr.sort.vcf
>$outdir/"$1"_"$2"_GATK_crossmap_hg38fr37.chr.sort.vcf.gz

$bgzip -c $outdir/"$1"_"$2"_GATK_crossmap_hg37fr38.chr.sort.vcf
>$outdir/"$1"_"$2"_GATK_crossmap_hg37fr38.chr.sort.vcf.gz

$tabix -p vcf $outdir/"$1"_"$2"_GATK_crossmap_hg38fr37.chr.sort.vcf.gz

$tabix -p vcf $outdir/"$1"_"$2"_GATK_crossmap_hg37fr38.chr.sort.vcf.gz

hg38_GIABsnvVCF=$outdir/"$1"_"$2"_GATK_crossmap_hg38fr37.chr.sort.vcf.gz

hg37_GIABsnvVCF=$outdir/"$1"_"$2"_GATK_crossmap_hg37fr38.chr.sort.vcf.gz

unset LD_LIBRARY_PATH


HCC_hg38_snvVCF=/dev/ngs003/bpan/reference_comparison/1_orignal_data/hg38/HG001_GRCh38_GI
AB_highconf_CG-IllFB-IllGATKHC-Ion-10X-SOLID_CHROM1-
X_v.3.3.2_highconf_PGandRTGphasetransfer.snv.recode.vcf.gz

HCC_hg37_snvVCF=/dev/ngs003/bpan/reference_comparison/1_orignal_data/hg37/HG001_GRCh37_GI
AB_highconf_CG-IllFB-IllGATKHC-Ion-10X-SOLID_CHROM1-
X_v.3.3.2_highconf_PGandRTGphasetransfer.snv.recode.chrAdd.vcf.gz

sdf_hg38=/dev/ngs003/bpan/reference_comparison/1_orignal_data/hg38/fasta/hg38sdf

sdf_hg37=/dev/ngs003/bpan/reference_comparison/1_orignal_data/hg37/fasta/hg37sdf_chrAdd

HCCbed_hg38=/dev/ngs003/bpan/reference_comparison/1_orignal_data/hg38/HG001_GRCh38_GIAB_
highconf_CG-IllFB-IllGATKHC-Ion-10X-SOLID_CHROM1-
X_v.3.3.2_highconf_nosomaticdel_noCENorHET7.bed

HCCbed_hg37=/dev/ngs003/bpan/reference_comparison/1_orignal_data/hg37/HG001_GRCh37_GIAB_
highconf_CG-IllFB-IllGATKHC-Ion-10X-SOLID_CHROM1-X_v.3.3.2_highconf_nosomaticdel.chrAdd.bed


outDirCmp_GATKHC_hg38=/dev/ngs005/scratch/hhong/GIAB/4_3_crossMap/comp_liftover_hg38_"$1"
_"$2"_GATK

outDirCmp_GATKHC_hg37=/dev/ngs005/scratch/hhong/GIAB/4_3_crossMap/comp_liftover_hg37_"$1"
_"$2"_GATK


java -Djava.io.tmpdir=$tempdir -Xmx20g -jar $RTG vcfeval \
```

```
    -b "${HCC_hg38_snvVCF}" \
    -c "${hg38_GIABsnvVCF}" \
    -o "${outDirCmp_GATKHC_hg38}" \
    -t "${sdf_hg38}"

java -Djava.io.tmpdir=$tempdir -Xmx20g -jar $RTG vcffilter \
    -i "${outDirCmp_GATKHC_hg38}"/tp.vcf.gz \
    --include-bed "${HCCbed_hg38}" \
    -o "${outDirCmp_GATKHC_hg38}"/tp_inhighconfbed.vcf.gz

java -Djava.io.tmpdir=$tempdir -Xmx20g -jar $RTG vcfeval \
    -b "${HCC_hg37_snvVCF}" \
    -c "${hg37_GIABsnvVCF}" \
    -o "${outDirCmp_GATKHC_hg37}" \
    -t "${sdf_hg37}"

java -Djava.io.tmpdir=$tempdir -Xmx20g -jar $RTG vcffilter \
    -i "${outDirCmp_GATKHC_hg37}"/tp.vcf.gz \
    --include-bed "${HCCbed_hg37}" \
    -o "${outDirCmp_GATKHC_hg37}"/tp_inhighconfbed.vcf.gz

gzip -d -c "${outDirCmp_GATKHC_hg38}"/tp_inhighconfbed.vcf.gz
>"${outDirCmp_GATKHC_hg38}"/tp_inhighconfbed.vcf

gzip -d -c "${outDirCmp_GATKHC_hg37}"/tp_inhighconfbed.vcf.gz
>"${outDirCmp_GATKHC_hg37}"/tp_inhighconfbed.vcf
```

```
cs_cal=/dev/ngs003/bpan/reference_comparison/scripts/cor_VCFvsVCF_v6_HCC_ATGCcnt.pl

perl $cs_cal "${outDirCmp_GATKHC_hg38}"/tp_inhighconfbed.vcf
$outdir/"$1"_"$2"_GATK_crossmap_hg38fr37.chr.sort.vcf ${hg38_vcf}
$outdir/CS_crossmap_"$1"_"$2"_GATK_hg38TFvshg38.stat.txt

perl $cs_cal "${outDirCmp_GATKHC_hg37}"/tp_inhighconfbed.vcf
$outdir/"$1"_"$2"_GATK_crossmap_hg37fr38.chr.sort.vcf ${hg37_vcf}
$outdir/CS_crossmap_"$1"_"$2"_GATK_hg37TFvshg37.stat.txt
```

```
######## (3) SNV comparison and statistic script ######################
#! /usr/bin/perl

use strict;
use warnings;

die "perl $0 <HCC_vcf> <VCF1_hg38fr37> <VCF2_hg38> <export>" if($#ARGV!=3);

my($hcc_hg38, $vcf1_hg38fr37, $vcf2_hg38, $export)=@ARGV;

my %recHCC=();
open(HCCFIL, $hcc_hg38) || die "$!";
while(<HCCFIL>){
        chomp;
        next if ($_=~/^#/);
        if($_=~/^chr/){
                $_=~s/^chr//;
        }
        my(@ary)=split/\t/, $_;
        $recHCC{$ary[0]}{$ary[1]}=();
}
my %basecnt=();
my %recH=();
my $varCnt=();
open(SNPFIL, $vcf1_hg38fr37) || die "$!";
```

```
while(<SNPFIL>){
	chomp;
#X	155674276	rs674707	G	A	50	PASS	callable=CS_HiSeqPE300xGATK_callable,CS_CGnormal_callable,CS_HiSeqPE300xfreebayes_callable;callsetnames=HiSeqPE300xGATK,CGnormal,HiSeqPE300xfreebayes,10XGATKhaplo,SolidPE50x50GATKHC,SolidSE75GATKHC;callsets=6;datasetnames=HiSeqPE300x,CGnormal,10XChromium,SolidPE50x50bp,SolidSE75bp;datasets=5;datasetsmissingcall=IonExome;filt=CS_SolidPE50x50GATKHC_filt;platformnames=Illumina,CG,10X,Solid;platforms=4	GT:AD:ADALL:DP:GQ:IGT:PS	1|1:97,365:0,294:652:99:1/1:PATMAT
#
	next if ($_=~/^#/);
	if($_=~/^chr/){
		$_=~s/^chr//;
	}
	$varCnt++;
	my(@arry)=split/\t/, $_;
	if($arry[8]!~/GT/){
		print $_."\tno GT info"."\n";
		next;
	}
	my $GT=();
	if($arry[9]=~/:/){
		($GT)=$arry[9]=~/(\S+?):/;
	}else{
		($GT)=$arry[9]=~/(\S+)/;
	}
	if(!defined $GT){
		print $_."\n";exit;
```

```
        }
        $recH{$arry[0]}{$arry[1]}=$arry[3]."\t".$arry[4]."\t".$GT;
        if(defined $basecnt{"0_pre"}{"$arry[3]"}){
            $basecnt{"0_pre"}{"$arry[3]"}++;
    }else{
        $basecnt{"0_pre"}{"$arry[3]"}=1;
    }
        if(exists $recHCC{$arry[0]}{$arry[1]}){
                if(defined $basecnt{"0_pre"}{'HCC'}){
                        $basecnt{"0_pre"}{'HCC'}++;
                }else{
                        $basecnt{"0_pre"}{'HCC'}=1;
                }
        }else{
                if(defined $basecnt{"0_pre"}{'LCC'}){
                        $basecnt{"0_pre"}{'LCC'}++;
                }else{
                        $basecnt{"0_pre"}{'LCC'}=1;
                }
        }
#       if($arry[6]=~/PASS/){
#               my $var=$arry[0]."_".$arry[1]."_".$arry[3]."_".$arry[4];
#               $recH{$var}=();
#       }
}
```

```
#my %basecnt=();
my $varCnt2=0;
my $csPosCnt=0;
my $csSNVCnt=0;
my $csGTCnt=0;
my $cntHCC=0; my $cntLCC=0;
open(FQFILE,$vcf2_hg38) || die "$!";
open(EXPORT,">$export") || die "$!";
while(<FQFILE>){
	chomp;
#chrX	151108239	.	C	T	50	PASS	platforms=3;platformnames=10X,Illumina,CG;datasets=3;datasetnames=10XChromium,HiSeqPE300x,CGnormal;callsets=4;callsetnames=10XGATKhaplo,HiSeqPE300xGATK,CGnormal,HiSeqPE300xfreebayes;datasetsmissingcall=IonExome,SolidPE50x50bp,SolidSE75bp;callable=CS_10XGATKhaplo_callable;filt=CS_SolidPE50x50GATKHC_filt;difficultregion=hg38_self_chain_withalts_gt10k	GT:DP:ADALL:AD:GQ:IGT:IPS:PS	0|1:746:196,213:0,0:99:0/1:.:PATMAT
	next if ($_=~/^#/);
	if($_=~/^chr/){
	 $_=~s/^chr//;
    }
	$varCnt2++;
	my(@arry2)=split/\t/, $_;
#	my $var2=$arry2[3]."_".$arry2[4];
	my($GT2)=$arry2[9]=~/(\S+?)\:/;
	if(exists $recH{$arry2[0]}{$arry2[1]}){
		$csPosCnt++;
```

```
#	if(defined $basecnt{"posCS"}{"$arry2[3]"}){
#		$basecnt{"posCS"}{"$arry2[3]"}++;
#	}else{
#		$basecnt{"posCS"}{"$arry2[3]"}=1;
#	}
	my(@snvgt)=split/\t/,$recH{$arry2[0]}{$arry2[1]};
	my($gt1,$gt2)=$snvgt[2]=~/(\d)\/(\d)/;
#		print $gt1."\t".$gt2."\n";exit;
	if(($snvgt[0] eq $arry2[3]) and ($snvgt[1] eq $arry2[4])){
		$csSNVCnt++;
		if(($gt1 eq $gt2) and ($snvgt[2] eq $GT2)){
			$csGTCnt++;
			if(defined $basecnt{"1_posCS_SNVCS"}{"$arry2[3]"}){
				$basecnt{"1_posCS_SNVCS"}{"$arry2[3]"}++;
			}else{
				$basecnt{"1_posCS_SNVCS"}{"$arry2[3]"}=1;
			}
			if(exists $recHCC{$arry2[0]}{$arry2[1]}){
				if(defined $basecnt{"1_posCS_SNVCS"}{'HCC'}){
					$basecnt{"1_posCS_SNVCS"}{'HCC'}++;
				}else{
					$basecnt{"1_posCS_SNVCS"}{'HCC'}=1;
				}
			}else{
				if(defined $basecnt{"1_posCS_SNVCS"}{'LCC'}){
```

```
                    $basecnt{"1_posCS_SNVCS"}{'LCC'}++;
                }else{
                    $basecnt{"1_posCS_SNVCS"}{'LCC'}=1;
                }
            }


        }else{
            my ($gtAlt)=$gt2.'/'.$gt1;
#           print $gtAlt."\n";exit;
            if(($snvgt[2] eq $GT2) or ($gtAlt eq $GT2)){
                $csGTCnt++;
                if(defined $basecnt{"1_posCS_SNVCS"}{"$arry2[3]"}){
                    $basecnt{"1_posCS_SNVCS"}{"$arry2[3]"}++;
                }else{
                    $basecnt{"1_posCS_SNVCS"}{"$arry2[3]"}=1;
                }
                if(exists $recHCC{$arry2[0]}{$arry2[1]}){
          if(defined $basecnt{"1_posCS_SNVCS"}{'HCC'}){
          $basecnt{"1_posCS_SNVCS"}{'HCC'}++;
         }else{
         $basecnt{"1_posCS_SNVCS"}{'HCC'}=1;
        }
        }else{
        if(defined $basecnt{"1_posCS_SNVCS"}{'LCC'}){
        $basecnt{"1_posCS_SNVCS"}{'LCC'}++;
```

```
            }else{
            $basecnt{"1_posCS_SNVCS"}{'LCC'}=1;
           }
}
              }else{
                        if(defined $basecnt{"2_posCS_SNVnot"}{"$arry2[3]"}){
                                $basecnt{"2_posCS_SNVnot"}{"$arry2[3]"}++;
                        }else{
                                $basecnt{"2_posCS_SNVnot"}{"$arry2[3]"}=1;
                        }
                        if(exists $recHCC{$arry2[0]}{$arry2[1]}){
        if(defined $basecnt{"2_posCS_SNVnot"}{'HCC'}){
            $basecnt{"2_posCS_SNVnot"}{'HCC'}++;
        }else{
            $basecnt{"2_posCS_SNVnot"}{'HCC'}=1;
        }
  }else{
        if(defined $basecnt{"2_posCS_SNVnot"}{'LCC'}){
            $basecnt{"2_posCS_SNVnot"}{'LCC'}++;
        }else{
            $basecnt{"2_posCS_SNVnot"}{'LCC'}=1;
        }
  }
              }
    }
```

```
                    }else{
                              if(defined $basecnt{"2_posCS_SNVnot"}{"$arry2[3]"}){
                          $basecnt{"2_posCS_SNVnot"}{"$arry2[3]"}++;
                }else{
                          $basecnt{"2_posCS_SNVnot"}{"$arry2[3]"}=1;
                    }
                              if(exists $recHCC{$arry2[0]}{$arry2[1]}){
                                        print "HCCbutNCSpos\t".$_."\n";
                     if(defined $basecnt{"2_posCS_SNVnot"}{'HCC'}){
                              $basecnt{"2_posCS_SNVnot"}{'HCC'}++;
                     }else{
                          $basecnt{"2_posCS_SNVnot"}{'HCC'}=1;
                     }
                 }else{
                     if(defined $basecnt{"2_posCS_SNVnot"}{'LCC'}){
                         $basecnt{"2_posCS_SNVnot"}{'LCC'}++;
                     }else{
                          $basecnt{"2_posCS_SNVnot"}{'LCC'}=1;
                     }
                }
                    }
                    delete $recH{$arry2[0]}{$arry2[1]};
          }
#                   else{
#                   if(defined $basecnt{"3_posNotCS"}{"$arry2[3]"}){
```

```
#                               $basecnt{"3_posNotCS"}{"$arry2[3]"}++;
#                       }else{
#                               $basecnt{"3_posNotCS"}{"$arry2[3]"}=1;
#                       }
#                       }
#           print EXPORT $_ . "\n";
#           else{
#                       print EXPORT "0"."\t".$_."\n";
#           }
}
close FQFILE;

foreach my $chrs (keys %recH){
            foreach my $poss(keys %{$recH{$chrs}}){
                        my(@arry3)=split/\t/, $recH{$chrs}{$poss};
                        if(defined $basecnt{"3_posNotCS"}{"$arry3[0]"}){
                                    $basecnt{"3_posNotCS"}{"$arry3[0]"}++;
                        }else{
                                    $basecnt{"3_posNotCS"}{"$arry3[0]"}=1;
                        }
                        if(exists $recHCC{$chrs}{$poss}){
                                    if(defined $basecnt{"3_posNotCS"}{'HCC'}){
                        $basecnt{"3_posNotCS"}{'HCC'}++;
                    }else{
                          $basecnt{"3_posNotCS"}{'HCC'}=1;
```

```
            }
        }else{
            if(defined $basecnt{"3_posNotCS"}{'LCC'}){
                $basecnt{"3_posNotCS"}{'LCC'}++;
            }else{
                $basecnt{"3_posNotCS"}{'LCC'}=1;
            }
        }
        }
}

#print EXPORT $varCnt."\t".$varCnt2."\t".$csPosCnt."\t".$csSNVCnt."\t".$csGTCnt;
#print EXPORT "\t".($csSNVCnt/$csPosCnt)."\t".($csGTCnt/$csPosCnt)."\t".($csGTCnt/$csPosCnt)."\n";
print EXPORT $varCnt;
#$varCnt2."\t".($varCnt-$csPosCnt)."\t".($csPosCnt-$csGTCnt);
#print EXPORT "\t".($csPosCnt/$varCnt)."\t".($csGTCnt/$varCnt)."\t".($csGTCnt/$csPosCnt);
print EXPORT "\t".$basecnt{'0_pre'}{'HCC'}."\t".$basecnt{'0_pre'}{'LCC'};
print EXPORT "\t".$basecnt{'0_pre'}{'A'}."\t".$basecnt{'0_pre'}{'T'};
print EXPORT "\t".$basecnt{'0_pre'}{'G'}."\t".$basecnt{'0_pre'}{'C'};
print EXPORT "\t".$varCnt2;
print EXPORT "\t".($basecnt{'3_posNotCS'}{'HCC'}+$basecnt{'3_posNotCS'}{'LCC'});
print EXPORT "\t".$basecnt{'3_posNotCS'}{'HCC'}."\t".$basecnt{'3_posNotCS'}{'LCC'};
print EXPORT "\t".$basecnt{'3_posNotCS'}{'A'}."\t".$basecnt{'3_posNotCS'}{'T'};
print EXPORT "\t".$basecnt{'3_posNotCS'}{'G'}."\t".$basecnt{'3_posNotCS'}{'C'};
print EXPORT "\t".($csPosCnt-$csGTCnt);
```

```
print EXPORT "\t".$basecnt{'2_posCS_SNVnot'}{'HCC'}."\t".$basecnt{'2_posCS_SNVnot'}{'LCC'};

print EXPORT "\t".$basecnt{'2_posCS_SNVnot'}{'A'}."\t".$basecnt{'2_posCS_SNVnot'}{'T'};

print EXPORT "\t".$basecnt{'2_posCS_SNVnot'}{'G'}."\t".$basecnt{'2_posCS_SNVnot'}{'C'};

print EXPORT "\n";

close EXPORT;

exit;
```
