# Supplementary material for: Similarities and differences between variants called with human reference genome HG19 or HG38
Source: BMC Bioinformatics. 2019 Mar 14;20(Suppl 2):101. doi: 10.1186/s12859-019-2620-0 (PMC6419332; doi:10.1186/s12859-019-2620-0)
Supplement: Supplementary file 3 — Supplemental figures. (PDF 900 kb) [file 12859_2019_2620_MOESM3_ESM.pdf]

Supplementary Figure 1-10:

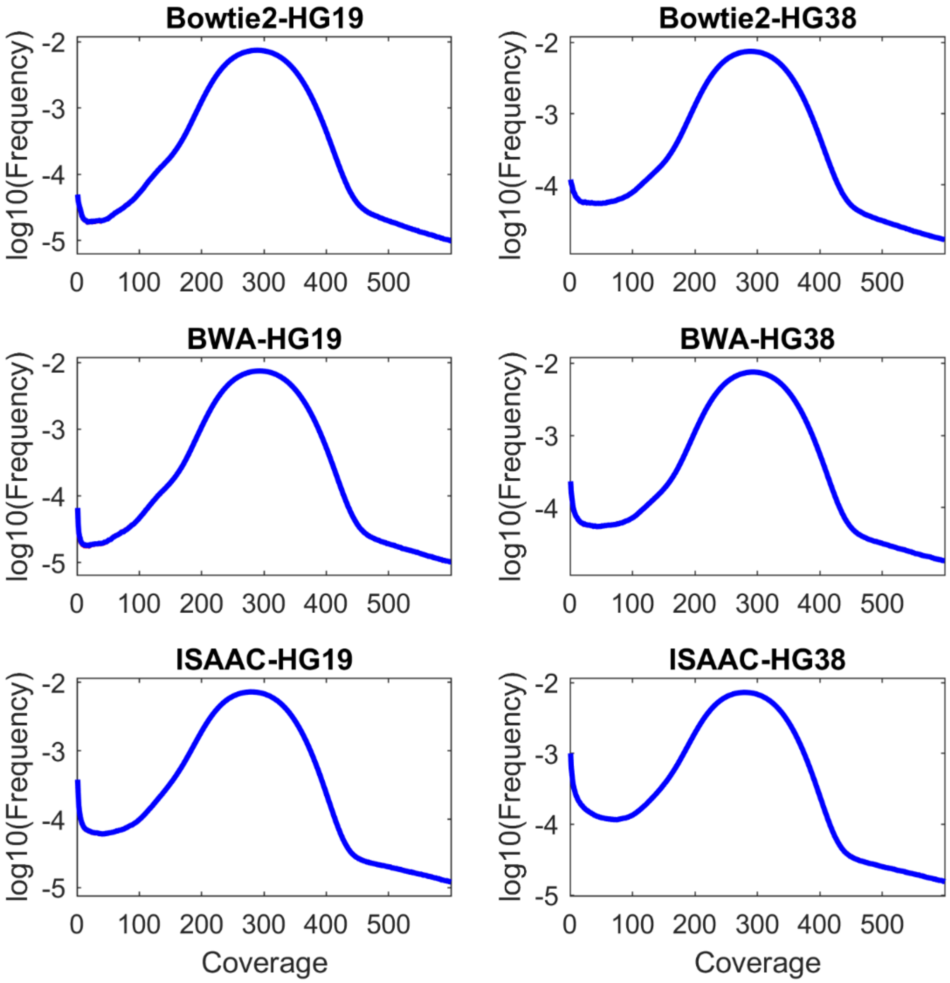

Supplement Figure S1. The frequency distributions of coverages for with or with-out GATK realignment. The number of bases that are covered by the same number of reads, coverage, indicated by the x-axis was given at the y-axis for alignments without GATK (red curve) and with GATK (blue curve). The two sub-figures in each row are statistic of alignment result from each aligner depicted by the titles above the sub-figures. The three sub-figures in the left panel are the three alignment results to reference HG19 while the right panel contains the alignment results to reference HG38.

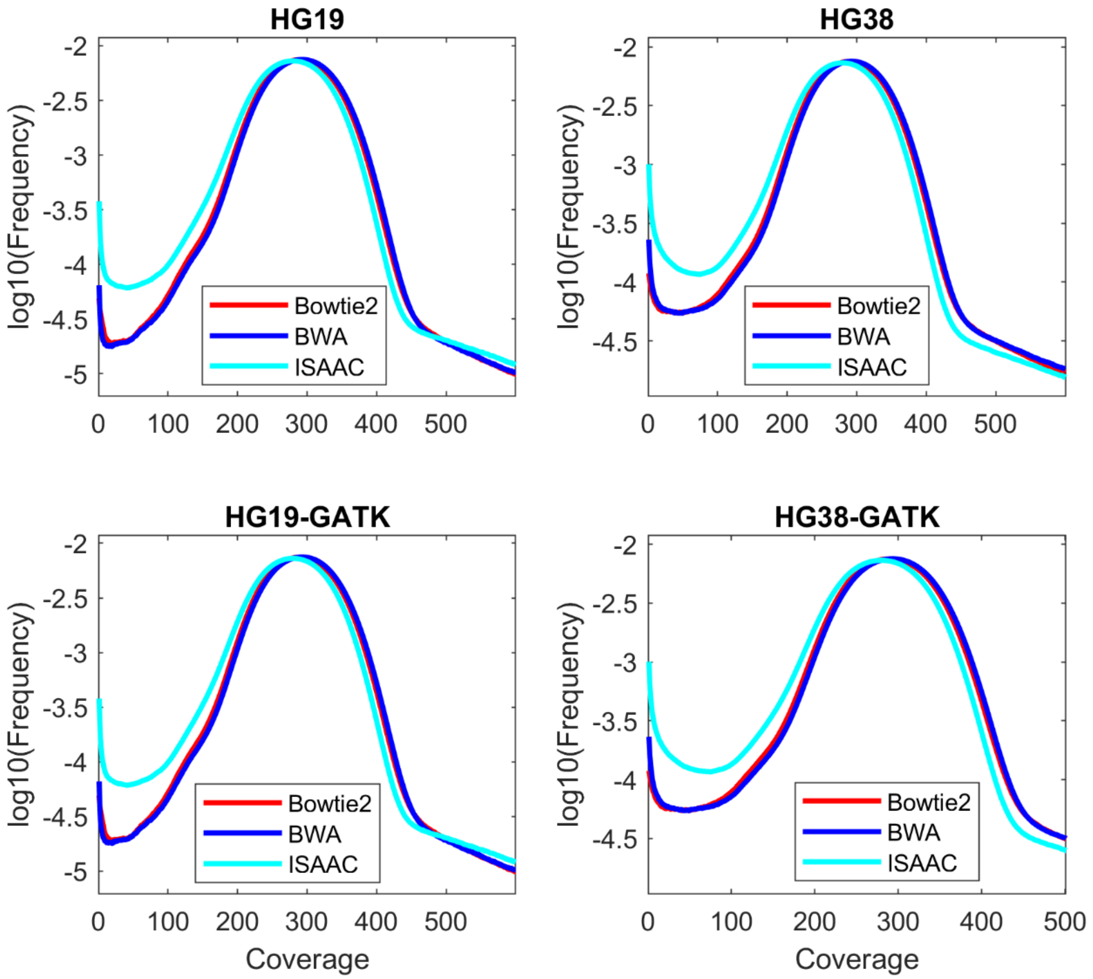

Supplement Figure S2. The distributions of coverage frequency for different aligners. The frequency of coverage indicated by the x-axis was given at the y-axis for different aligners, Bowtie2 (red curve), BWA (blue curve) and ISAAC (cyan curve). The two sub-figures in each row are statistic of alignment result without GATK realignment depicted by the titles above the sub-figures. The two sub-figures in the left panel are the alignment results to reference HG19 while the right panel contains the alignment results to reference HG38.

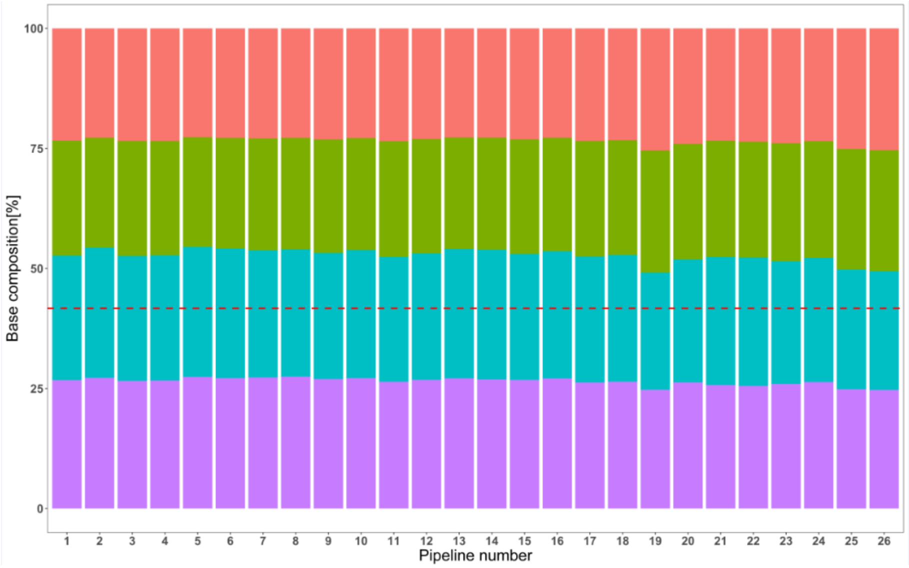

Supplement Figure S3. Base compositions of reference alleles for the position-discordant SNVs from HG19 comparison and Picard conversion. The base composition for discordant SNVs from each pipeline indicated by the x-axis was given at the y-axis for different aligners, “A”, “T”, “G”, and “C” (boxes from top to bottom). The normal GC content in the genome is marked in the figure by the red dotted line parallel to the x-axis. The x-axis gives pipeline numbers that could be found in Table 1. The y-axis depicts the conversion rates.

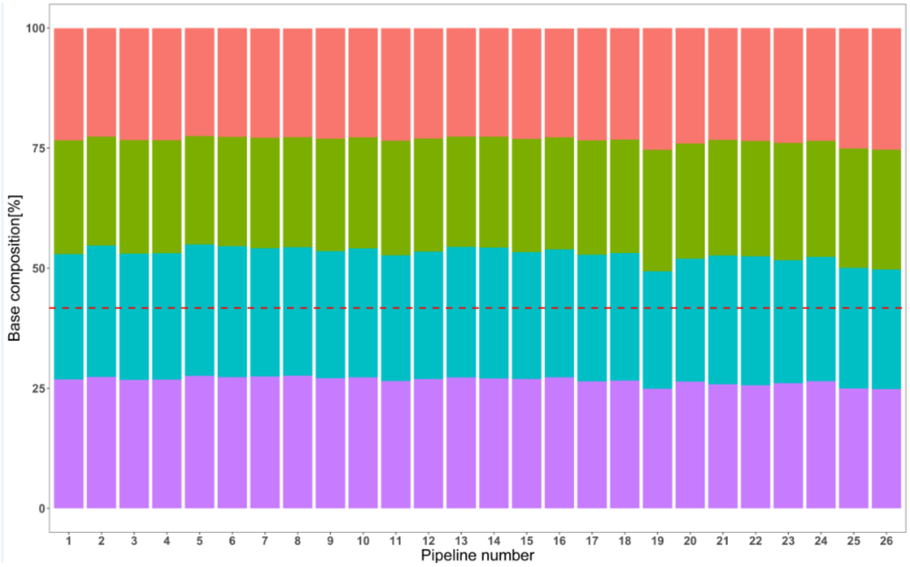

Supplement Figure S4. Base compositions of reference alleles for the position-discordant SNVs from HG19 comparison and CrossMap conversion. The base composition for discordant SNVs from each pipeline indicated by the x-axis was given at the y-axis for different aligners, “A”, “T”, “G”, and “C” (boxes from top to bottom). The normal GC content in the genome is marked in the figure by the red dotted line parallel to the x-axis. The x-axis gives pipeline numbers that could be found in Table 1. The y-axis depicts the conversion rates.

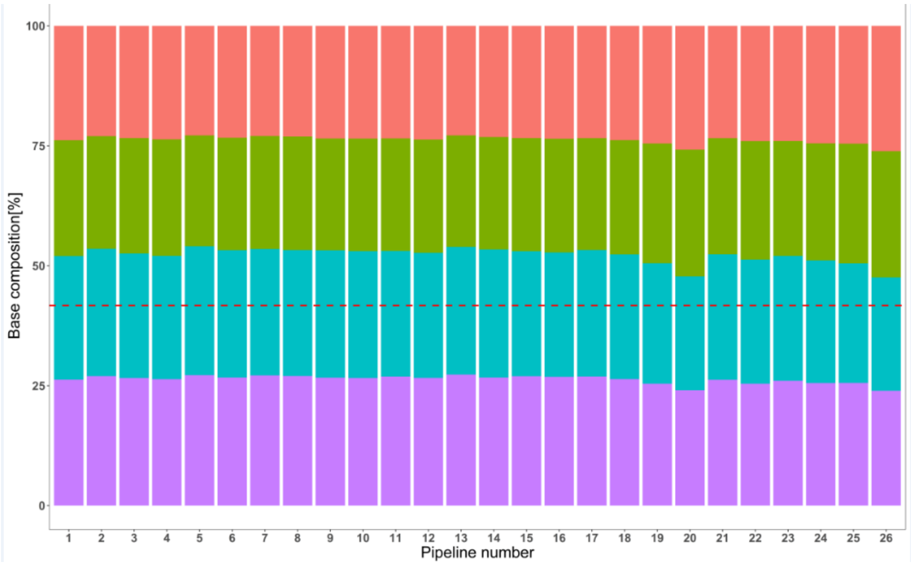

Supplement Figure S5. Base compositions of reference alleles for the position-discordant SNVs from HG38 comparison and Picard conversion. The base composition for discordant SNVs from each pipeline indicated by the x-axis was given at the y-axis for different aligners, "A", "T", "G", and "C" (boxes from top to bottom). The normal GC content in the genome is marked in the figure by the red dotted line parallel to the x-axis. The x-axis gives pipeline numbers that could be found in Table 1. The y-axis depicts the conversion rates.

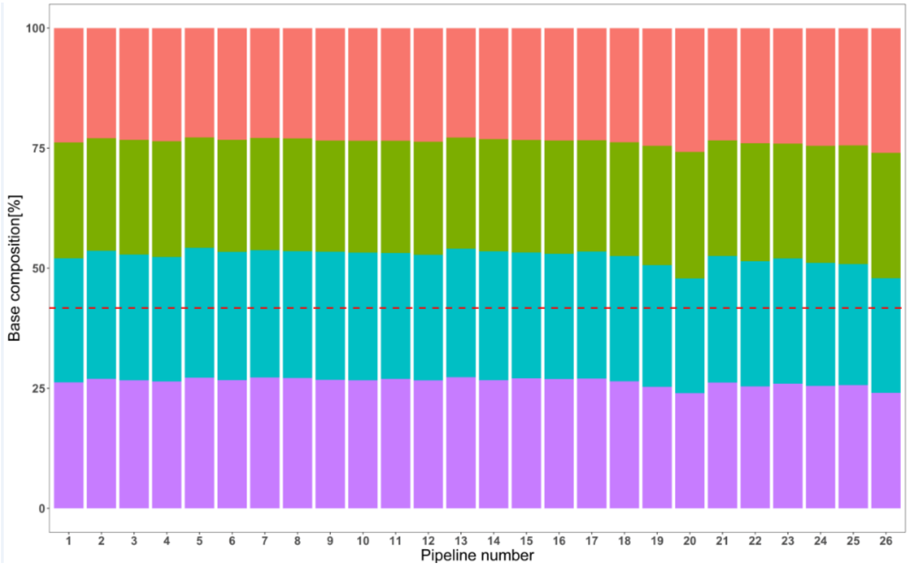

Supplement Figure S6. Base compositions of reference alleles for the position-discordant SNVs from HG38 comparison and CrossMap conversion. The base composition for discordant SNVs from each pipeline indicated by the x-axis was given at the y-axis for different aligners, "A", "T", "G", and "C" (boxes from top to bottom). The normal GC content in the genome is marked in the figure by the red dotted line parallel to the x-axis. The x-axis gives pipeline numbers that could be found in Table 1. The y-axis depicts the conversion rates.

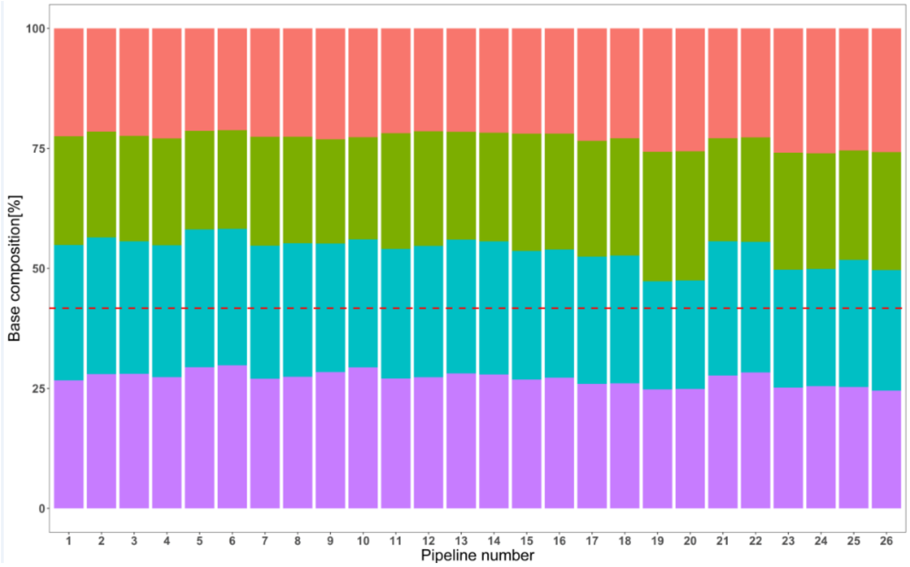

Supplement Figure S7. Base compositions of reference alleles for the genotype-discordant SNVs from HG19 comparison and Picard conversion. The base composition for discordant SNVs from each pipeline indicated by the x-axis was given at the y-axis for different aligners, "A", "T", "G", and "C" (boxes from top to bottom). The normal GC content in the genome is marked in the figure by the red dotted line parallel to the x-axis. The x-axis gives pipeline numbers that could be found in Table 1. The y-axis depicts the conversion rates.

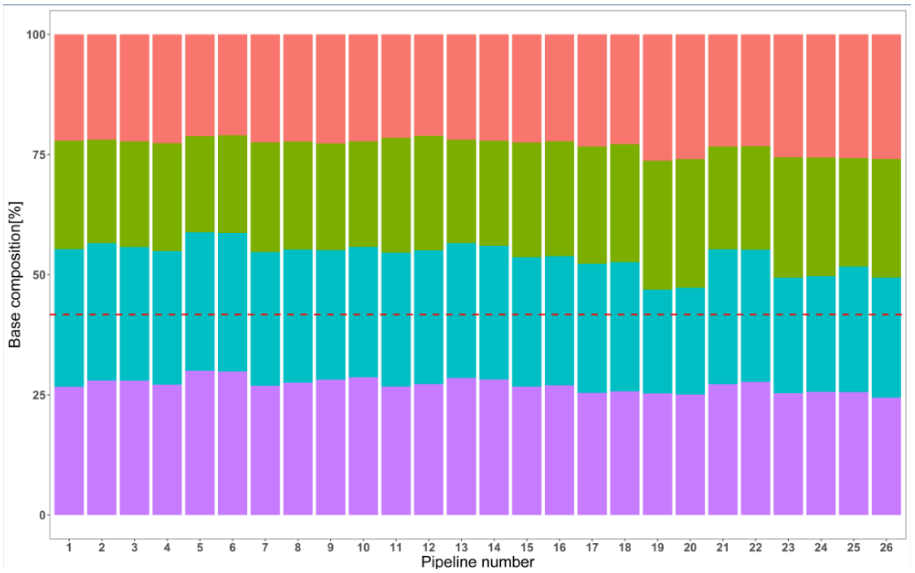

Supplement Figure S8. Base compositions of reference alleles for the genotype-discordant SNVs from HG19 comparison and CrossMap conversion. The base composition for discordant SNVs from each pipeline indicated by the x-axis was given at the y-axis for different aligners, "A", "T", "G", and "C" (boxes from top to bottom). The normal GC content in the genome is marked in the figure by the red dotted line parallel to the x-axis. The x-axis gives pipeline numbers that could be found in Table 1. The y-axis depicts the conversion rates.

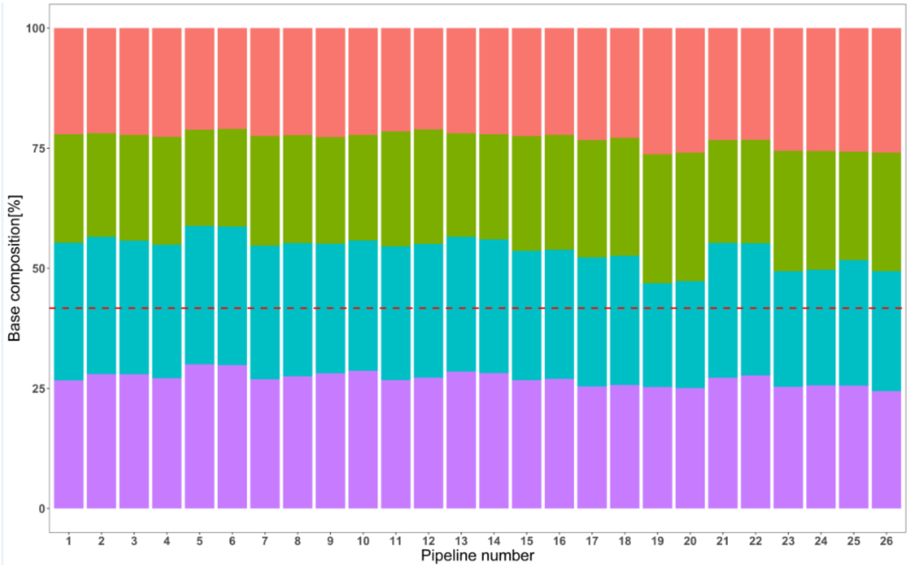

Supplement Figure S9. Base compositions of reference alleles for the genotype-discordant SNVs from HG38 comparison and Picard conversion. The base composition for discordant SNVs from each pipeline indicated by the x-axis was given at the y-axis for different aligners, “A”, “T”, “G”, and “C” (boxes from top to bottom). The normal GC content in the genome is marked in the figure by the red dotted line parallel to the x-axis. The x-axis gives pipeline numbers that could be found in Table 1. The y-axis depicts the conversion rates.

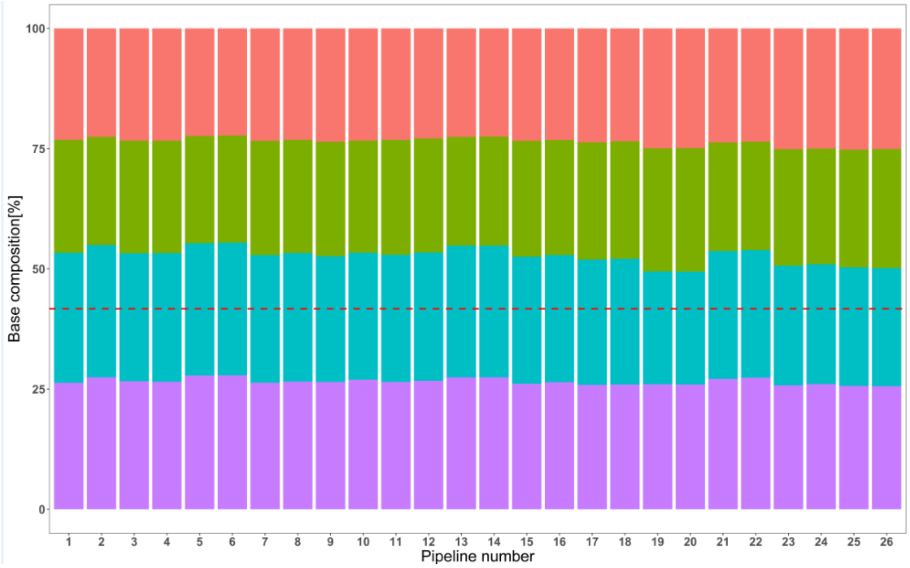

Supplement Figure S10. Base compositions of reference alleles for the genotype-discordant SNVs from HG38 comparison and CrossMap conversion. The base composition for discordant SNVs from each pipeline indicated by the x-axis was given at the y-axis for different aligners, “A”, “T”, “G”, and “C” (boxes from top to bottom). The normal GC content in the genome is marked in the figure by the red dotted line parallel to the x-axis. The x-axis gives pipeline numbers that could be found in Table 1. The y-axis depicts the conversion rates.
